# pH‐Activatable Pre‐Nanozyme Mediated H_2_S Delivery for Endo‐Exogenous Regulation of Oxidative Stress in Acute Kidney Injury

**DOI:** 10.1002/advs.202303901

**Published:** 2024-03-06

**Authors:** Wei Jiang, Xinyue Hou, Yuanbo Qi, Zhigang Wang, Ying Liu, Xuejiao J. Gao, Tingting Wu, Jiancheng Guo, Kelong Fan, Wenjun Shang

**Affiliations:** ^1^ Department of Kidney Transplantation The First Affiliated Hospital of Zhengzhou University Zhengzhou 450001 China; ^2^ Nanozyme Medical Center School of Basic Medical Sciences Academy of Medical Science Zhengzhou University Zhengzhou 450001 China; ^3^ College of Chemistry and Chemical Engineering Jiangxi Normal University Nanchang 330022 P. R. China; ^4^ CAS Engineering Laboratory for Nanozyme Key Laboratory of Protein and Peptide Pharmaceuticals Institute of Biophysics Chinese Academy of Sciences Beijing 100101 China; ^5^ University of Chinese Academy of Sciences Beijing 101408 China

**Keywords:** acute kidney injury, antioxidant, H_2_S, pre‐nanozyme, pH‐responsive

## Abstract

Oxidative stress induced by excess reactive oxygen species (ROS) is a primary pathogenic cause of acute kidney injury (AKI). Development of an effective antioxidation system to mitigate oxidative stress for alleviating AKI remains to be investigated. This study presents the synthesis of an ultra‐small Platinum (Pt) sulfur cluster (Pt_5.65_S), which functions as a pH‐activatable prefabricated nanozyme (pre‐nanozyme). This pre‐nanozyme releases hydrogen sulfide (H_2_S) and transforms into a nanozyme (Ptzyme) that mimics various antioxidant enzymes, including superoxide dismutase and catalase, within the inflammatory microenvironment. Notably, the Pt_5.65_S pre‐nanozyme exhibits an endo‐exogenous synergy‐enhanced antioxidant therapeutic mechanism. The Ptzyme reduces oxidative damage and inflammation, while the released H_2_S gas promotes proneurogenesis by activating Nrf2 and upregulating the expression of antioxidant molecules and enzymes. Consequently, the Pt_5.65_S pre‐nanozyme shows cytoprotective effects against ROS/reactive nitrogen species (RNS)‐mediated damage at remarkably low doses, significantly improving treatment efficacy in mouse models of kidney ischemia‐reperfusion injury and cisplatin‐induced AKI. Based on these findings, the H_2_S‐generating pre‐nanozyme may represent a promising therapeutic strategy for mitigating inflammatory diseases such as AKI and others.

## Graphical abstract

This study presents a novel ultrasmall Pt_5.65_S pre‐nanozyme that releases hydrogen sulfide and exhibits inducible antioxidant enzyme‐like activity in an acidic and inflammatory microenvironment for managing acute kidney injury by promoting Nrf2 activation and mitigating oxidative damage.

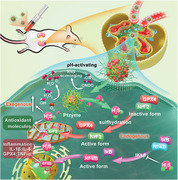

## 1 Introduction

Acute kidney injury (AKI), a life‐threatening clinical condition characterized by a rapid decline in renal function, has emerged as a significant global health concern with high mortality and morbidity rates. Recent clinical studies have revealed that patients with AKI face a markedly increased risk of developing chronic kidney disease and end‐stage renal disease primarily due to the absence of effective pharmacological interventions to accelerate renal recovery.^[^ ^1^, ^2^ ^]^ Ischemia‐reperfusion injury (IRI) and chemotherapy are major factors contributing to clinical AKI, which is associated with the development of a systemic inflammatory response mediated by enhanced reactive oxygen species (ROS) production and elevated oxidative stress.^[^ ^1^, ^3^, ^4^ ^]^ Consequently, one of the primary approaches to treating AKI involves efficiently eliminating excessive ROS from the kidney to regulate the renal microenvironment and effectively avoid or mitigate extensive oxidative damage. *N*‐acetyl cysteine and acetyl‐*L*‐carnitine represent two examples of broad‐spectrum ROS‐scavenging antioxidants that are thought to be optimal AKI treatment options. However, the widespread clinical utilization of these antioxidants is hindered by their limited bioavailability and restricted capacity to scavenge ROS.^[^ ^5^, ^6^ ^]^

In biological systems, highly reactive oxygen‐containing compounds are referred to as ROS, which participate in numerous clinical and physiological processes. The primary constituents of ROS include the highly reactive hydroxyl radical (•OH), singlet oxygen (^1^O_2_), hydrogen peroxide (H_2_O_2_), and superoxide anion radical (O_2_^•−^), who engage in various signaling pathways and actively support physiological activities such as signal transduction, immune responses, and cellular function regulation.^[^ ^7^, ^8^, ^9^, ^10^ ^]^ The delicate equilibrium of ROS generation and elimination is maintained under normal physiological conditions. Antioxidants, including catalase (CAT), superoxide dismutase (SOD), ascorbate, and glutathione (GSH), effectively scavenge redundant ROS. However, under pathological circumstances, the capacity of organism to eliminate surplus ROS may be compromised.^[^ ^11^ ^]^ The excessive production of ROS can disrupt in vivo redox balance, triggering an oxidative stress response in healthy cells. Persistent oxidative stress can result in irreversible damage to DNA, proteins, and lipids, leading to various physiological disorders, such as cancer and inflammation.^[^ ^1^, ^12^ ^]^ In 2007, Yan group discovered that Fe_3_O_4_ nanoparticles exhibit enzyme‐like activity.^[^ ^13^ ^]^ These characteristics make nanoparticles effective mimics of cellular antioxidant enzymes, such as SOD, CAT, and glutathione peroxides (GPX4), and offer new possibilities for treating oxidative stress‐related disorders.^[^ ^14^ ^]^ Nanozymes, characterized by their unique nano‐size, low production cost, ease of modification, and high stability, have garnered increasing interest in biology and nanomedical sciences. With advancements in nanotechnology, V_2_O_5_ nanowires exhibit strong ROS scavenging capabilities in kidney, neuronal, prostate, and cervical cells. According to Mugesh et al., these nanowires mimic GPX4 and possess the ability to fully restore redox balance without interfering with cellular antioxidant defenses, providing invaluable protection against harmful oxidative damage to biomolecules.^[^ ^15^ ^]^ However, their efficacy is significantly constrained by off‐target toxicities stemming from “always‐on” enzyme‐like activities and reduced catalytic efficiencies due to substrate inaccessibility to catalytic centers resulting from steric hindrance. Furthermore, nanozymes solely scavenging free radicals through an exogenous single pathway often prove inadequate. Consequently, developing nanozymes with conditionally activatable catalytic activity and employing multiple‐pathway synergistic enhancement strategies may be scientifically important in treating and preventing kidney diseases associated with ROS/reactive nitrogen species (RNS).^[^ ^16^, ^17^ ^]^

Hydrogen sulfide (H_2_S), also known as sulfane, stands as the simplest sulfur‐containing molecule and represents the third constituent of the gaseotransmitter ensemble in mammalian systems, following nitric oxide (NO) and carbon monoxide (CO)^[^ ^18^, ^19^ ^]^ H_2_S exerts its influence on protein activity through a post‐translational modification process termed over‐sulfidation, thereby impacting cellular function.^[^ ^20^ ^]^ Functioning as an antioxidant, H_2_S facilitates ATP synthesis and counteracts free radicals, thus preventing the oxidation of protein function induced by lipid peroxidation.^[^ ^21^, ^22^ ^]^ Extensive research highlights the pivotal role of H_2_S in regulating glomerular filtration rate, sodium reabsorption, renin release, and oxygen sensing within the renal system, establishing it as an indispensable regulator of vascular and cellular function.^[^ ^21^, ^23^ ^]^ Thus, it is reasonable to propose that H_2_S play an essential role in the treatment of AKI and even preserving renal function in procedures such as nephrectomy and renal transplantation.

**Scheme 1:**
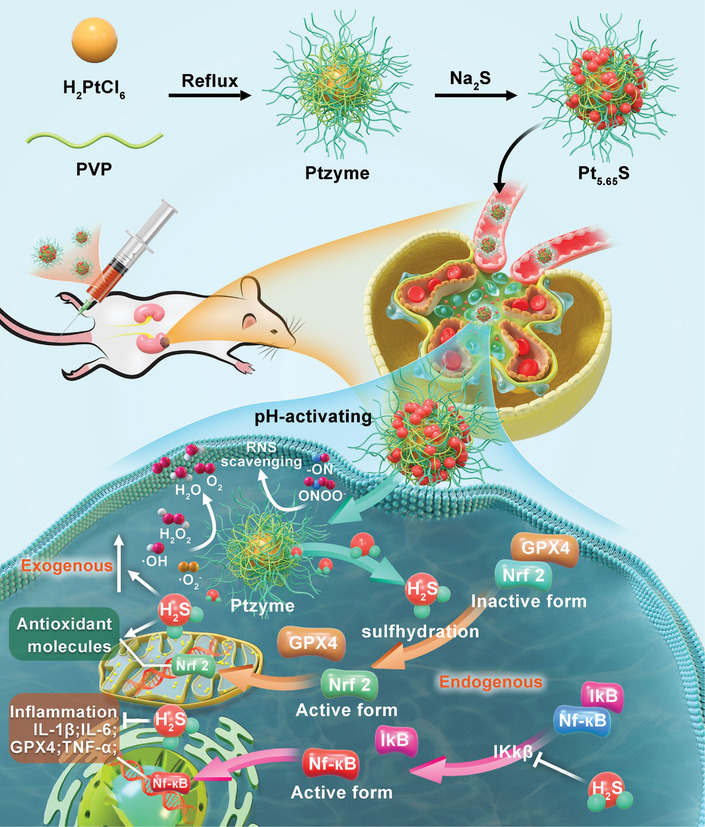
Schematic illustration of Pt_5.65_S pre‐nanozyme and its therapeutic mechanisms on inflammation‐associated kidney disease based on endogenous and exogenous regulation of oxidative stress.

In this study, we design an ultra‐small platinum‐based pre‐nanozyme (Pt_5.65_S) through the reaction of Pt cluster nanozyme (Ptzyme) with Na_2_S, which exhibits pH‐activated exceptional broad‐spectrum ROS/RNS scavenging capabilities, excellent biosafety, and highly efficient renal clearance to effectively treat and prevent inflammation‐related kidney diseases (**Scheme** **1**). Notably, the Pt_5.65_S pre‐nanozyme exhibits minimal enzyme‐like activity in normal physiological environments, while it generates H_2_S gas in acidic and inflammatory settings, mimicking the natural antioxidant system and functioning as a CAT and SOD. The pre‐nanozyme (Pt_5.65_S) efficiently regulates oxidative stress in the kidney in mouse models of IRI and cisplatin‐induced AKI. By sulfating NF‐κB and inhibiting the Ikκβ enzyme, H_2_S restricts the activation of the NF‐κB pathway and prevents the translocation of NF‐κB into the nucleus, thereby averting a cytokine storm. Additionally, H_2_S enhances the expression of antioxidant molecules and enzymes and promotes Nrf2 activation. Thus, a synergistic approach involving H_2_S gas molecules and nanozymes can be employed to achieve endo‐exogenous synergy‐enhanced antioxidant therapeutic effects. Due to its extremely small size, the Pt_5.65_S pre‐nanozyme accumulates and is cleared in the kidney. Furthermore, Pt_5.65_S pre‐nanozyme demonstrates sustained long‐term biosafety in vivo, as evidenced by the biochemical indices in mice. Consequently, this study presents an effective method for treating IRI and AKI by imitating the antioxidant enzymatic activity of the Pt_5.65_S pre‐nanozyme.

## 2 Results and Discussion

### 2.1 The Synthesis and Characterization of Pt_5.65_S Pre‐Nanozyme

**Figure 1:**
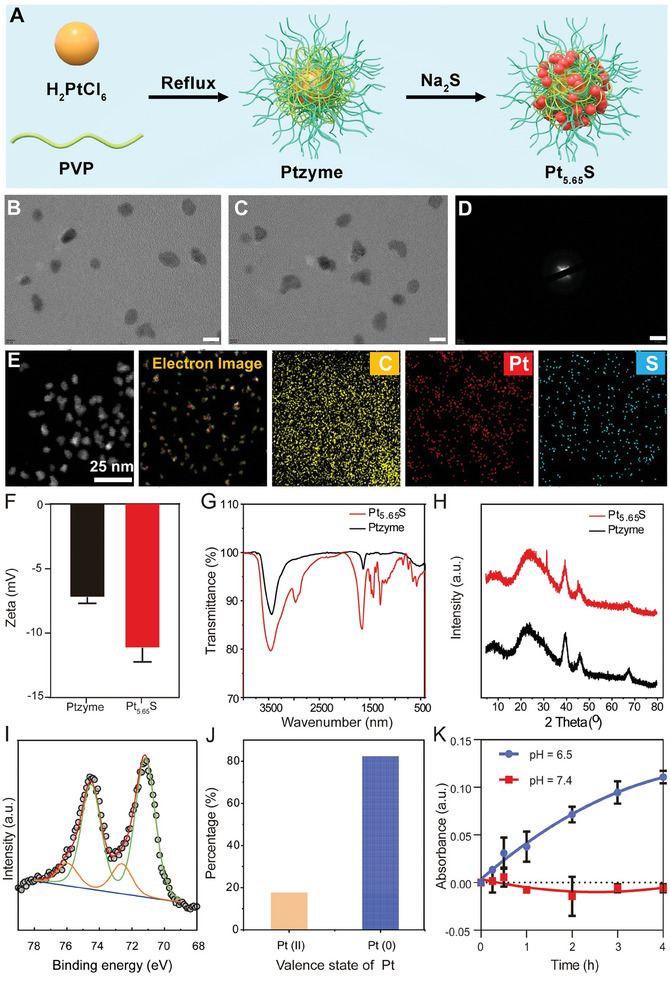
Preparation and characterization of Pt_5.65_S pre‐nanozyme. A) Schematic preparation of Pt_5.65_S pre‐nanozyme. B) TEM image of Ptzyme (Scale bar = 5 nm). C) TEM image of Pt_5.65_S pre‐nanozyme (Scale bar = 5 nm). D) SAED pattern of Pt_5.65_S pre‐nanozyme (Scale bar = 5 nm^−1^). E) Elemental mapping of Pt_5.65_S pre‐nanozyme. F) Zeta potential of Ptzyme and Pt_5.65_S pre‐nanozyme. G) FTIR of Ptzyme and Pt_5.65_S pre‐nanozyme. H) PXRD of Ptzyme and Pt_5.65_S pre‐nanozyme. I) XPS of Pt_5.65_S pre‐nanozyme. J) Content of Pt^II^ and Pt^0^ in the Pt_5.65_S pre‐nanozyme. K) Release of H_2_S from Pt_5.65_S pre‐nanozyme at pH 6.5 and pH 7.4. *n* = 3, data represent means ± SD.

Pt_5.65_S pre‐nanozyme was successfully synthesized using physical adsorption and ligand interaction approaches, which are rapid, reproducible, and environmentally friendly methods (**Figure** **1**A). Transmission electron microscopy (TEM) images demonstrated that the Pt_5.65_S pre‐nanozyme exhibits a uniformly spherical nanoparticle morphology, with an average particle size of 4 nm, meeting the glomerular filtration threshold of ≈3.25 nm^[^ ^24^ ^]^ (Figure 1B,C). Figure 1D shows the selected area electron diffraction (SAED) pattern of the Pt_5.65_S pre‐nanozyme, indicating that the nanozyme complex is amorphous, which is consistent with the results of X‐ray Diffraction (XRD) analysis shown in Figure 1H. Furthermore, elemental mapping of Pt_5.65_S pre‐nanozyme confirmed the presence of Pt and S signals in the composite, providing solid evidence that Ptzyme was successfully reacted with Na_2_S (Figure 1E). Dynamic light scattering (DLS) analysis revealed that Ptzymes and Pt_5.65_S pre‐nanozyme exhibited hydrodynamic particle sizes of 16.73 ± 3.5 and 14.45 ± 2.613 nm, respectively, indicating that Na_2_S reacting only slightly affects the size distribution of Ptzyme (Figure S2, Supporting Information). In addition, Na_2_S reacting reduced the mean zeta potential of Ptzymes from ≈−7.312 to ≈−11.23 mV (Figure 1F). The successful reaction of Na_2_S with Ptzymes was confirmed through fourier transform infrared (FT‐IR) and X‐ray diffraction (XRD) analysis, as depicted in Figure 1G,H. The emergence of fresh peaks in the XRD and FT‐IR spectra of Pt_5.65_S pre‐nanozyme, as opposed to Ptzyme, validates the effective incorporation of S^2−^ into the composite. The surface chemistry of Pt_5.65_S pre‐nanozyme was analyzed through X‐ray photoelectron spectroscopy (XPS), presenting visible absorption peaks of Pt elements (Pt for Pt_5.65_S), as demonstrated in Figure 1I. High‐resolution XPS spectra of Pt orbits showed peaks corresponding to Pt 4f7/2 at binding energies of 72.6 (Pt^II^) and 71.2 (Pt^0^) eV. The mass fractions of Pt and PtS were calculated based on the peak area of Pt 4f7/2, indicating that the proportions of Pt and PtS were ≈17.7% and 82.3%, respectively (Figure 1J). Importantly, the production of H_2_S from Pt_5.65_S pre‐nanozyme was then evaluated, as show in Figure 1K almost no release of H_2_S was detected in the neutral microenvironment (pH = 7.4), whereas a sustained H_2_S production could be observed in the acid environment (pH 6.5). Taken together, the resulting ultra‐small pH‐conditioned activated pre‐nanozyme was successfully synthesized, denoted as Pt_5.65_S pre‐nanozyme.

### 2.2 Oxygen/Nitrogen Radicals Scavenging Scavenging by Pt_5.65_S Pre‐Nanozyme

**Figure 2:**
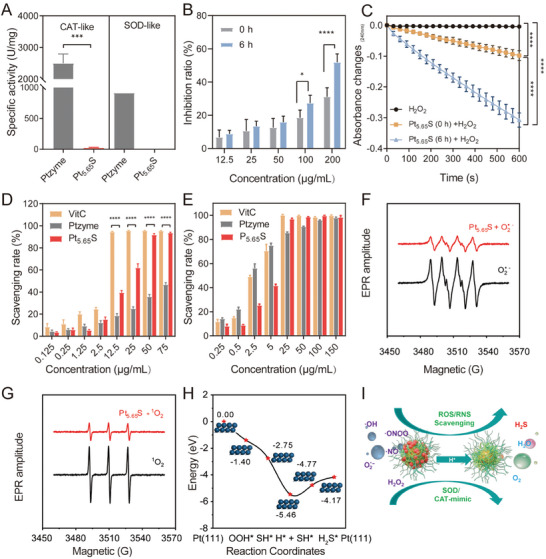
ROS scavenging capability analyses of Pt_5.65_S pre‐nanozyme. A) The CAT/SOD‐like activities of Ptzyme and Pt_5.65_S pre‐nanozyme in buffer solution (pH 7.4). B) The SOD‐like activities of Pt_5.65_S pre‐ nanozyme after incubation for different times in buffer solution (pH 6.5). C) The CAT‐like activities of Pt_5.65_S pre‐nanozyme after incubation for different times in buffer solution (pH 6.5). D) DPPH^•^ scavenging rate of Vitamin C (Vit C), Ptzyme, and Pt_5.65_S pre‐nanozyme. E) ABTS^•+^ scavenging rate of Vit C, Ptzyme, and Pt_5.65_S pre‐nanozyme. F,G) Pt_5.65_S pre‐nanozyme reducing the generation of superoxide radical (·O_2_^−^) and singlet oxygen (^1^O_2_), illustrated by ESR spectroscopy. H) The energy profiles in eV for the competitive adsorption of S^2−^ and O_2_^•−^ on the Pt(111) surface and the generation of gaseous H_2_S under acidic conditions. I) Schematic illustration of Pt_5.65_S pre‐nanozyme pH active antioxidative activities and release of H_2_S. The outcomes were compared via one‐way ANOVA (with Tukey's post hoc correction for multiple comparisons). *n* = 3, data represent means ± SD, ns represents no statistical difference, ^*^ *p* < 0.1, ^***^ *p* < 0.001, ^****^ *p* < 0.0001.

Ptzyme exhibited excellent SOD/CAT‐mimic activities under normal physiological environment (**Figure** **2**A). However, upon reacting with Na_2_S, Pt_5.65_S pre‐nanozyme presented low SOD/CAT‐like activities in phosphate buffer at pH 7.4, as illustrated in Figure 2A. Intriguingly, when incubated in an acidic environment with the release of hydrogen sulfide, Pt_5.65_S pre‐nanozyme activity gradually increased with the increasing of Ptzyme transformation, as demonstrated in Figure 2B,C. These results imply that Pt_5.65_S pre‐nanozyme mimics the SOD/CAT cascade activity of natural enzymes and functions more effectively as an antioxidant in the inflammatory microenvironment. Using 2,2‐azino‐bis‐(3‐ethylbenzothiazoline‐6‐sulfonate) (ABTS) and 1,1‐diphenyl‐2‐picrylhydrazyl (DPPH) free radical scavenging assays, the capacity of Ptzyme and Pt_5.65_S pre‐nanozyme to scavenge nitrogen radicals was further investigated. Both Ptzyme and Pt_5.65_S pre‐nanozyme showed significant suppression of ABTS and DPPH free radical generation (ABTS^•+^ and DPPH^•^), as depicted in Figure 2D,E, and Figure S3 (Supporting Information), indicating enhanced antioxidant activity. Additionally, Pt_5.65_S pre‐nanozyme exhibited a significant reduction in the formation of O_2_^•−^ by the xanthine‐xanthine oxidase (X‐XOD) system, as observed by electron spin resonance (ESR) spectroscopy (Figure 2F). Owing to their SOD and CAT‐like activity, Pt_5.65_S pre‐nanozyme scavenged ^1^O_2_, resulting in a decrease in the formation of ^1^O_2_ from the TiO_2_ photocatalytic system (Figure 2G).

To comprehend the mechanism by which S^2−^ ions can inhibit the SOD‐like activity of the Pt_5.65_S nanomaterial, DFT calculations were employed to examine the reaction processes and the corresponding energy profile for the competitive adsorption of S^2−^ and O_2_^•−^ on Pt(111) surface and the generation of gaseous H_2_S under acidic condition. Figure 2H presents the results of our study, which demonstrate that the adsorption energy of O_2_^•−^ was −1.40 eV under acidic conditions, resulting in the formation of OOH* (* denotes the adsorption state). Subsequently, we evaluated the competitive adsorption of S^2−^ at the OOH* adsorption site and observed that this was an exothermic process that generated HS*. The more negative adsorption energy of S^2−^ (−2.75 eV) in comparison to O_2_^•−^ 1.40 eV) contributed to this outcome. Consequently, our findings indicate that S^2−^ exhibited greater affinity toward the surface of Pt(111) and could competitively replace the active site of O_2_^•−^ that ultimately decreased the SOD‐like activity of the material. Subsequently, H^+^ could be readily adsorbed on the Pt(111) surface, accompanied by an energy drop of 2.71 eV. The conversion of H* and HS* to H_2_S* could be achieved with a modest energy rise of 0.69 eV, which could be easily accomplished at room temperature. Ultimately, the adsorbed H_2_S* desorbed from the Pt(111) surface with a desorption energy of 0.60 eV, resulting in the production of gaseous H_2_S. These observations signify that S^2−^ could be converted to H_2_S at the surface of Pt(111) in an acidic environment. Furthermore, our findings align with the experimental evidence demonstrating that S^2−^ could impede SOD‐like activity by competitively occupying the active site. Overall, our study indicates that Pt_5.65_S pre‐nanozyme could transform into nanozyme (Ptzyme) and effectively mimic the SOD/CAT cascade activities of natural enzymes (Figure 2I). As antioxidants, they possess the potential to alleviate inflammation‐associated kidney diseases.

### 2.3 Cell‐Protective Activities of Pt_5.65_S Pre‐Nanozyme In Vitro

**Figure 3:**
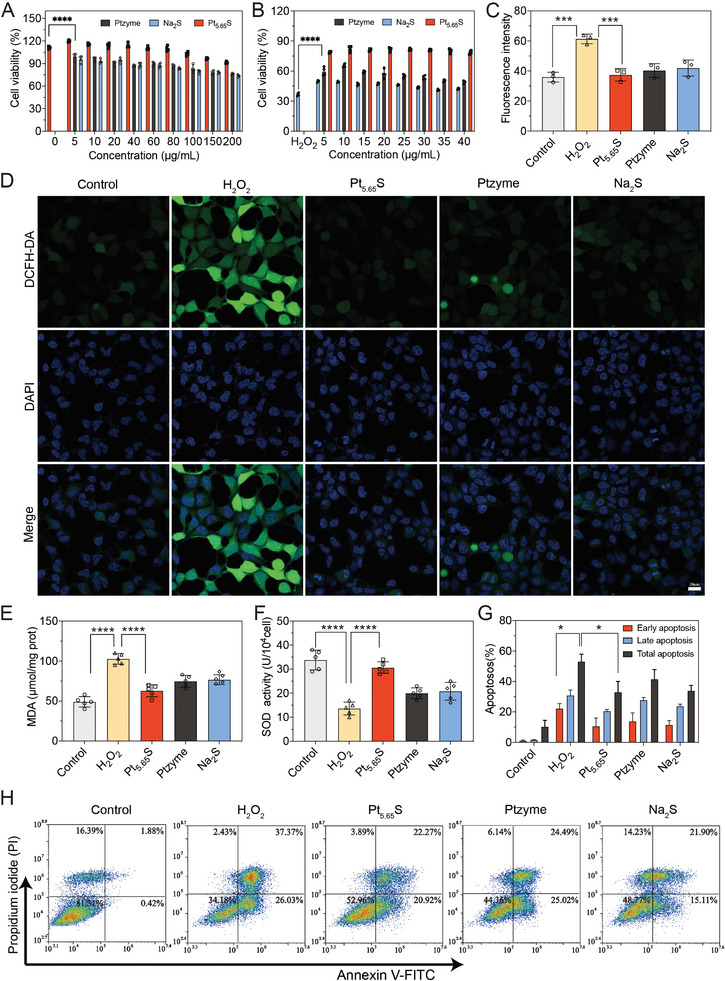
In vitro antioxidant and anti‐inflammatory properties of Pt_5.65_S pre‐nanozyme. A) The cell viability analyses of HEK293 after incubation with Ptzyme, Na_2_S, and Pt_5.65_S pre‐nanozyme for 24 h. B) Cell viability analyses of H_2_O_2_‐stimulated HEK293 cells after co‐culture with Ptzyme, Na_2_S, and Pt_5.65_S pre‐nanozyme for 24 h. C) Semi‐quantitative analyses of intracellular ROS in Figure 3D. D) Intracellular ROS detection of H_2_O_2_‐stimulated HEK293 cells after being treated with Ptzyme, Na_2_S, and Pt_5.65_S pre‐nanozyme for 24 h was observed by fluorescent microscopy. Scale bar = 20 µm. The analyses of E) MDA and F) SOD, in H_2_O_2_‐stimulated HEK293 cells after treatment with Ptzyme, Na_2_S, and Pt_5.65_S pre‐nanozyme for 24 h. (*n* = 5, data represent means ± SD, ns represents no statistical difference, ^***^ *p* < 0.001, ^****^ *p* < 0.0001). G) Semi‐quantitative analyses of flow cytometry in flowjo. H) The apoptosis in each group was assessed by flow cytometry (a. Control, b. H_2_O_2_, c. Pt_5.65_S pre‐nanozyme, d. Ptzyme, e. Na_2_S). The outcomes were compared via one‐way ANOVA (with Tukey's post hoc correction for multiple comparisons). *n* = 3, data represent means ± SD, ns represents no statistical difference, ^***^ *p* < 0.001, ^****^ *p* < 0.0001.

Considering that oxidative stress plays a significant role in the pathogenesis of AKI, we first examined the potential effects of Pt_5.65_S pre‐nanozyme on H_2_O_2_‐induced oxidative stress in vitro. Initially, we assessed the biocompatibility of Pt_5.65_S pre‐nanozyme through hemolysis assay and incubating various concentrations of Ptzyme, Na_2_S, and Pt_5.65_S pre‐nanozyme (0 – 200 µg mL^−1^) with pre‐cultured human embryonic kidney 293 (HEK293) cells in 96‐well plates for 24 h, respectively. The positive control of red blood cells (RBCs) lysis was effectively induced by ddH_2_O, whereas treatment with PBS, Pt_5.65_S pre‐nanozyme, and Ptzyme did not result in any apparent hemolysis, as evidenced by the clear supernatant following centrifugation (Figure S4, Supporting Information). The CCK‐8 assay results demonstrated that Pt_5.65_S pre‐nanozyme was well‐tolerated by the cells, with over 80% of cells surviving at dosages up to 200 µg mL^−1^ for 24 h (**Figure** **3**A). Subsequently, we exposed HEK293 cells to H_2_O_2_ to examine the antioxidant capacity of Pt_5.65_S pre‐nanozyme in vitro. After being treated with different amounts of Pt_5.65_S pre‐nanozyme for 60 min, HEK293 cells were stimulated with 200 µm H_2_O_2_ for 24 h. The CCK‐8 results revealed that ≈60% of cells died after treatment with H_2_O_2_ alone, while a low concentration (5 µg mL^−1^) of Pt_5.65_S pre‐nanozyme significantly improved cell viability compared to the other two interventions (Figure 3B).

As the release of intracellular lactate dehydrogenase (LDH) into the culture media indicates irreversible cell death caused by cell membrane disruption, we performed an LDH release assay to determine the cell‐protective activity of Pt_5.65_S pre‐nanozyme against H_2_O_2_‐induced cell death. Figure S5A (Supporting Information) demonstrated that Pt_5.65_S pre‐nanozyme significantly inhibited H_2_O_2_‐induced LDH release as well. To further investigate whether Pt_5.65_S pre‐nanozyme protected HEK293 cells by reducing ROS, we determined intracellular ROS levels using a cell‐permeable DCFH‐DA sensor. As depicted in Figure 3C,D, pretreatment with Ptzyme, Na_2_S, or Pt_5.65_S pre‐nanozyme substantially reduced ROS production in H_2_O_2_‐stimulated HEK293 cells, with Pt_5.65_S pre‐nanozyme demonstrating a stronger ability to prevent ROS formation compared to the others.

An excess of ROS can induce lipid peroxidation, which plays a critical role in cell death, including apoptosis, autophagy, and ferroptosis. Superoxide dismutase (SOD) is one of the primary antioxidant enzymes that protect cells from harmful ROS. Figure 3E,F showed that Pt_5.65_S pre‐nanozyme pretreatment decreased the lipid peroxidation product malondialdehyde (MDA) and increased SOD levels in H_2_O_2_‐treated cells. Moreover, Pt_5.65_S pre‐nanozyme notably upregulated the mRNA expression of GPX4 (Figure S5B, Supporting Information) and enhanced the ratio of reduced glutataione (GSH) to its oxidized product glutathione disulfide (GSSG) (Figure S5C, Supporting Information) in vitro. Additionally, we investigated the effect of Pt_5.65_S pre‐nanozyme on total lipid peroxidation in cells using the BODIPY probe. The results indicated that H_2_O_2_ treatment in HEK293 cells caused a shift in BODIPY 581/591C11 fluorescence from red (reduced) to green (oxidized), which was significantly reversed by Pt_5.65_S pre‐nanozyme treatment (Figure S5D, Supporting Information)

Research has indicated that excessive production and accumulation of ROS can lead to oxidative stress, triggering the activation of proinflammatory signaling pathways and the release of various proinflammatory cytokines. This ultimately leads to a decrease in the local pH value. Given that Pt_5.65_S pre‐nanozyme exhibited the ability to release H_2_S gas in an acidic environment, we assessed the anti‐inflammatory properties of Pt_5.65_S pre‐nanozyme in vitro. As anticipated, treatment with Pt_5.65_S pre‐nanozyme resulted in a significant reduction in the mRNA levels of three key proinflammatory cytokines, namely *IL‐6*, *IL‐1β*, and *TNF‐α*, in comparison to the H_2_O_2_‐treated group. Ptzyme and Na_2_S also showed some inhibition of *IL‐6*, *IL‐1β*, and *TNF‐α* mRNA production, but were less effective than Pt_5.65_S pre‐nanozyme (Figure S5E–G, Supporting Information). Considering ROS plays a crucial role in the activation of apoptosis, we further evaluated the anti‐apoptosis effect of Pt_5.65_S using Annexin V‐FITC/PI double labeling in vitro. As depicted in Figure 3G,H, apoptotic cells constituted ≈63.4% of the H_2_O_2_ group, while the apoptotic rate could be significantly reduced (to ≈20%) through Pt_5.65_S pre‐nanozyme treatment. These findings suggest that Pt_5.65_S pre‐nanozyme enhances the protective effects of Ptzyme or Na_2_S against oxidative stress‐induced cell death.

**Figure 4:**
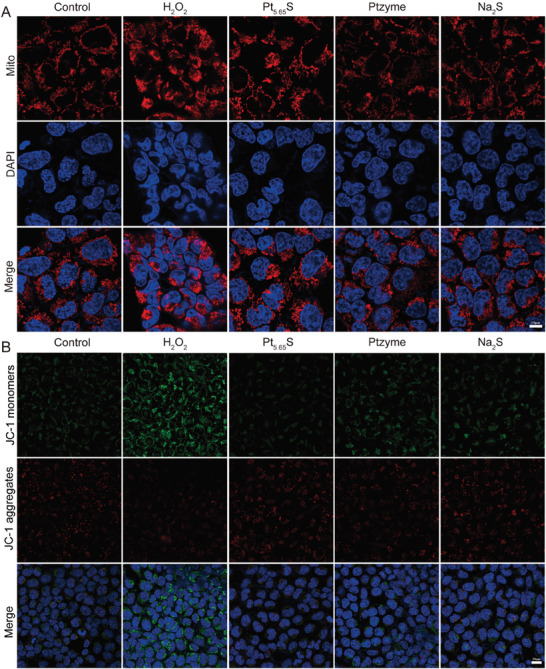
In vitro antioxidant and anti‐inflammatory properties of Pt_5.65_S pre‐nanozyme. A) Representative micrographs display MitoTracker staining in HEK293 cells. Scale bar = 10 µm. B) Mitochondrial Δψm values in the indicated treatment groups were assessed using confocal microscopy. scale bar = 20 µm.

The primary source of intracellular ROS production is the mitochondria, and elevated levels of intracellular ROS can, in turn, impair mitochondrial architecture and function. Changes in mitochondrial morphology have been considered an early indicator of ROS‐induced mitochondrial dysfunction; therefore, we investigated the potential effects of Pt_5.65_S pre‐nanozyme on mitochondrial morphology using the red‐fluorescent dye MitoTracker. Our results demonstrated that treatment with Pt_5.65_S pre‐nanozyme significantly inhibited H_2_O_2_‐induced mitochondrial fragmentation (**Figure** **4**A). We further assessed ROS‐induced mitochondrial lipid oxidation using MitoSOX labeling, and Figure S6 (Supporting Information) showed that Pt_5.65_S pre‐nanozyme substantially reduced H_2_O_2_‐induced mitochondrial ROS production in HEK293 cells. Since mitochondrial membrane potential (ΔΨm) is a vital indicator of mitochondrial function, we employed the JC‐1 fluorescent probe to evaluate changes in ΔΨm. When the ΔΨm is high, JC‐1 accumulates in the mitochondrial matrix and emits red fluorescence. Figure 4B revealed that ΔΨm levels were notably low in HEK293 cells after H_2_O_2_ incubation, which is in agreement with previous research. However, in the Pt_5.65_S pre‐nanozyme treatment group, the JC‐1 red/green fluorescence intensity ratio was higher compared to the Ptzyme or Na_2_S treated groups, indicating the superior ability of Pt_5.65_S pre‐nanozyme to maintain normal ΔΨm under oxidative stress. These findings suggest that Pt_5.65_S pre‐nanozyme has a significant protective effect on mitochondrial morphology and function. Consequently, the activated SOD/CAT‐like Pt_5.65_S pre‐nanozyme exhibits demonstrates exceptional antioxidant and anti‐inflammatory activities in vitro, indicating that Pt_5.65_S pre‐nanozyme may have potential therapeutic applications in the prevention of AKI.

### 2.4 Protective Effects of Pt_5.65_S Pre‐Nanozyme on Ischemia‐Reperfusion‐Induced AKI

**Figure 5:**
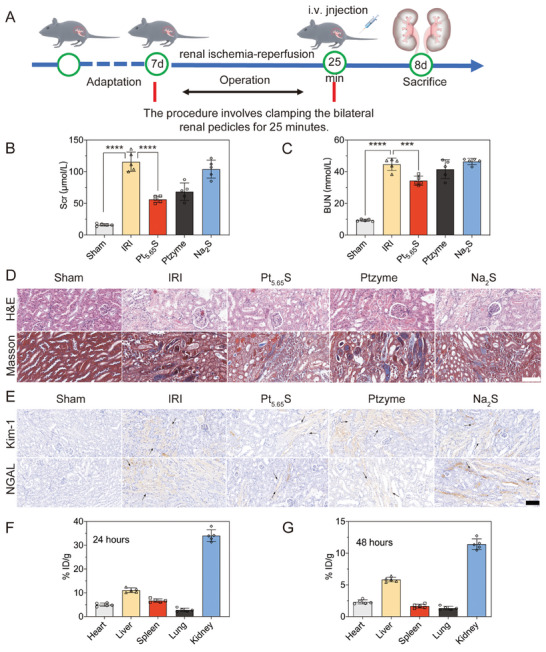
Antioxidant properties of Pt_5.65_S pre‐nanozyme in vivo. A) Operation of renal ischemia‐reperfusion in mice. B) The level of serum creatinine (Scr) in the serum of each group. C) The level of urea nitrogen (BUN) in the serum of each group. D) Haematoxylin and Eosin (H&E), Masson staining analyses of the kidneys from Sham, IRI, Pt_5.65_S pre‐nanozyme, Ptzyme, and Na_2_S treatment groups. Scale bar = 100 µm. E) Immunohistochemical staining analyses of the Kim‐1, NGAL (all brown) on the kidney tissues from IRI mice after different treatments. The black arrow indicates the site of inflammation and damage in the kidneys. Scale bar = 100 µm. Biodistribution of Pt per gram of tissues in the main tissues (heart, liver, spleen, lung, and kidney) of IRI mice after injection of 15 mg kg^−1^ Pt_5.65_S pre‐nanozyme by intravenous administration at F) 24 h and G) 48 h. The samples were filtered and measured by ICP‐MS. The outcomes were compared using one‐way ANOVA (with Tukey's post hoc correction for multiple comparisons). *n* = 5, data represent means ± SD, ns represents no statistical difference, ^***^ *p* < 0.001, ^****^ *p* < 0.0001.

The exceptional antioxidant and anti‐inflammatory properties of Pt_5.65_S pre‐nanozyme, along with its promising biosafety in vitro, led us to investigate whether it could alleviate AKI in vivo. We first examined the therapeutic effects of Pt_5.65_S pre‐nanozyme on IRI‐AKI by establishing a renal IRI mouse model, as depicted in **Figure** **5**A. In brief, a small artery clip was employed to clamp the bilateral renal arteries for 25 min, inducing ischemia followed by reperfusion injury. After 24 h, the IRI and sham‐operated mice were euthanized. Serum creatinine (Scr) and blood urea nitrogen (BUN) concentrations were measured to evaluate kidney function. As demonstrated in Figure 5B,C, IRI mice exhibited significantly increased Scr and BUN values, indicating the successful development of the ischemia‐reperfusion‐induced IRI mouse model. Kidney function results also revealed that Pt_5.65_S pre‐nanozyme treatment substantially reduced Scr and BUN levels in IRI mice.

Additionally, kidney damage was assessed through histologic staining with hematoxylin and eosin (H&E) and Masson's trichrome. Figure 5D illustrated that IRI led to severe tubular injuries and expanded interstitial areas, while Pt_5.65_S pre‐nanozyme treatment notably mitigated these kidney injuries. Markedly decreased expression levels of kidney damage indicators, such as kidney injury molecule‐1 (Kim‐1) and neutrophil gelatinase‐associated lipocalin (NGAL), were also observed in IRI mice following Pt_5.65_S pre‐nanozyme treatment (Figure 5E). Subsequently, we evaluated the biodistribution of Pt_5.65_S pre‐nanozyme in IRI mice. The Pt content in various organs such as kidney, liver, heart, spleen, and lung at different time points accurately reflects the in vivo distribution of Pt_5.65_S pre‐nanozyme. This is attributed to the distinctive Pt element present in Pt_5.65_S pre‐nanozyme. Utilizing ICP‐MS analysis for Pt quantification, the distribution of Pt_5.65_S pre‐nanozyme in the kidney reached 34.12% ID/g at 24 hours post‐injection, gradually decreasing to 11.56% ID/g after 48 hours. Notably, the accumulation of Pt in the kidney surpassed that in other organs, as illustrated in Figure 5F,G. We noted that the mononuclear phagocyte system sequesters the majority of the injected nanomaterials. The Pt_5.65_S pre‐nanozyme, with a size of ∼3.25 nm, exhibited low liver accumulation but significant renal accumulation (mononuclear phagocyte system, MPS). However, the Pt_5.65_S pre‐nanozyme formulation could effectively slow down the in vivo rapid absorption and clearance of nanoparticles by the MPS. Considering that renal injury induced by ischemia‐reperfusion can potentially lead to mild hepatic damage, we conducted a supplementary analysis by assessing the activity of aspartate transaminase (AST) and alanine transaminase (ALT), two widely recognized markers of liver injury in clinical diagnostics. Our findings revealed a significant reduction in AST and ALT levels following pretreatment with Pt_5.65_S pre‐nanozyme, aligning with the ability of liver to absorb these nanomaterials (Figure S7, Supporting Information). These results provide further evidence of the effective mitigation of oxidative damage in mice with renal IRI through the utilization of Pt_5.65_S pre‐nanozyme formulation.

**Figure 6:**
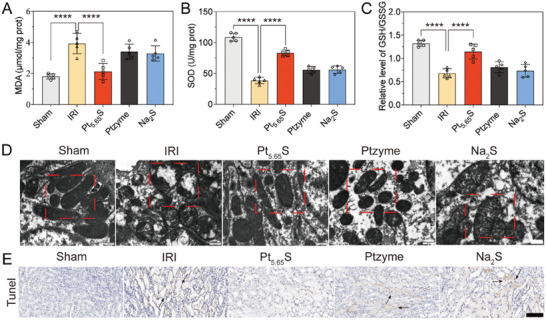
Anti‐inflammatory properties of Pt_5.65_S pre‐nanozyme in vivo. A) The analyses level of the level of MDA in the kidneys of each group. B) The analyses of the level of SOD in the kidneys of each group. C) The analyses of the level of GSH/GSSG in the kidneys of each group. D) Representative TEM images of mitochondria in the kidney from each group. The red box in the figure depicts representative TEM images showing the morphology of mitochondria under different conditions. Scale bar = 500 nm. E) TUNEL staining analyses of the kidneys from different groups. The black arrows indicate areas of damaged kidney cells Scale bar = 100 µm. The outcomes were compared using one‐way ANOVA (with Tukey's post hoc correction for multiple comparisons). *n* = 5, data represent means ± SD, ns represents no statistical difference, ^***^p < 0.001, ^****^p < 0.0001.

During the progression of renal IRI, large quantities of oxygen free radicals are produced by neutrophils and vascular endothelial cells. This process stimulates leukocyte activation and the release of inflammatory cytokines through xanthine oxidase and mitochondria, which in turn triggers an amplification effect in the inflammatory cascade, severely compromising normal renal functions. Ischemia diminishes the activity of the antioxidant enzyme SOD and the production of GSH levels, impacting the mitochondrial antioxidant system and rendering cells more susceptible to oxidative damage. As a consequence of lipid peroxidation, breakdown products such as MDA are released, serving as a kidney injury marker and an indirect indicator of ROS. SOD, the primary enzyme in the antioxidant enzyme defense system, can halt the free radical chain reaction leading to lipid peroxidation and protect cells and tissues from ROS damage. As demonstrated in **Figure** **6**A–C, IRI significantly elevated peroxidized lipid MDA levels while suppressing SOD activity and GSH levels. However, the Pt_5.65_S pre‐nanozyme (Figure 6B,C) effectively increased SOD activities and GSH levels and reduced MDA contents (Figure 6A).

Mitochondrial morphology is highly sensitive to oxidative stress, as mitochondria serve as both a source and a target for ROS. Mice subjected to IRI and treated with Ptzyme or Na_2_S exhibited some degree of abnormal mitochondrial morphology. However, transmission electron microscopy (TEM) images revealed that the mitochondrial structure in the kidneys of Pt_5.65_S pre‐nanozyme‐treated IRI mice was nearly normal, with no evident deformation or loss of cristae (Figure 6D). These findings were in line with the results of in vitro Mito‐tracker and JC‐1 staining assays of the Pt_5.65_S pre‐nanozyme, as displayed in Figure 4A,B. Furthermore, Pt_5.65_S pre‐nanozyme could significantly decreased Caspase‐3 expression (Figure S8, Supporting Information) and alleviated renal tubular apoptosis, as assessed by TUNEL (Terminal deoxynucleotidyl transferase‐mediated dUTP Nick‐End Labeling) staining in IRI mice (Figure 6E), since ROS and mitochondria play crucial roles in apoptosis activation. These results indicate that the combination of Ptzyme with modified Na_2_S hydrogen sulfide gas release (Pt_5.65_S pre‐nanozyme group) significantly enhanced therapeutic efficacy. This improvement was attributed to the prolonged circulation of nanozymes, their selective accumulation in the kidneys, and the reduced generation of lipid ROS. Consequently, oxidative stress played a vital role in the development of ischemia‐reperfusion‐induced AKI in mice, and Pt_5.65_S pre‐nanozyme functioned as an effective antioxidant.

### 2.5 Therapeutic Mechanisms of Pt_5.65_S Pre‐Nanozyme on IRI

**Figure 7:**
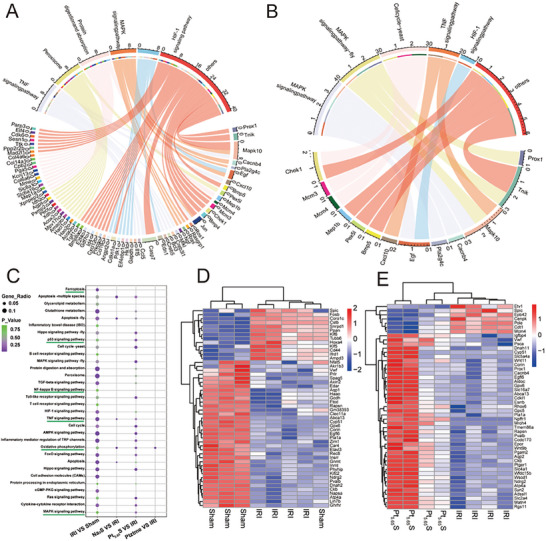
Therapeutic mechanisms of Pt_5.65_S pre‐nanozyme on IRI. A) Enriched chord diagram of the KEGG pathways based on the intersection of functional genes. B) KEGG pathway‐rich chord map in IRI VS Pt_5.65_S group based on intersection functional genes. C) The KEGG pathways enrichment analysis of the DEGs between Sham, IRI, Ptzyme, Na_2_S, and Pt_5.65_S pre‐nanozyme groups with the significantly enriched pathways. D) Heatmap diagram of transcriptomic profiles of the differentially expressed genes between Sham and IRI groups. E) Heatmap diagram of transcriptomic profiles of the differentially expressed genes between IRI and Pt_5.65_S pre‐nanozyme groups.

To explore the underlying therapeutic mechanisms of Pt_5.65_S pre‐nanozyme in alleviating kidney IRI, we performed RNA sequencing. The Pt_5.65_S pre‐nanozyme treatment reduced oxidative stress‐induced inflammation, as demonstrated by significant alterations in gene enrichment associated with positive regulation of defense response, ferroptosis, cytokine‐cytokine receptor interaction, and NF‐κB pathways according to the Encyclopedia of Genes and Genomes (KEGG) results (**Figure** **7**A,B). KEGG analysis of the top pathways revealed inflammation‐related pathways, which were individually reported in Figure 7C. The edgeR analysis identified 1214 genes with significant differences between the IRI and Sham groups, indicating the effectiveness of the IRI model (Figure S9, Supporting Information). We identified functional genes related to inflammation through a comparison to gene sets, followed by intersection analysis and KEGG analysis, which showed significant changes in genes involved in Nrf2 pathway, NF‐κB signaling, and the TNF signaling pathway. Additionally, to further identify Nrf2 antioxidant target genes and signaling pathways related to renal protection, we evaluated the functionality of Pt_5.65_S pre‐nanozyme by comparing it with Nrf2 and ferroptosis gene sets (Figure 7D,E). This was followed by cross‐analysis and KEGG assessment, indicating gene functional enrichment. These findings suggest that the Pt_5.65_S pre‐nanozyme acts as an inflammation regulator in the management of IRI.

**Figure 8:**
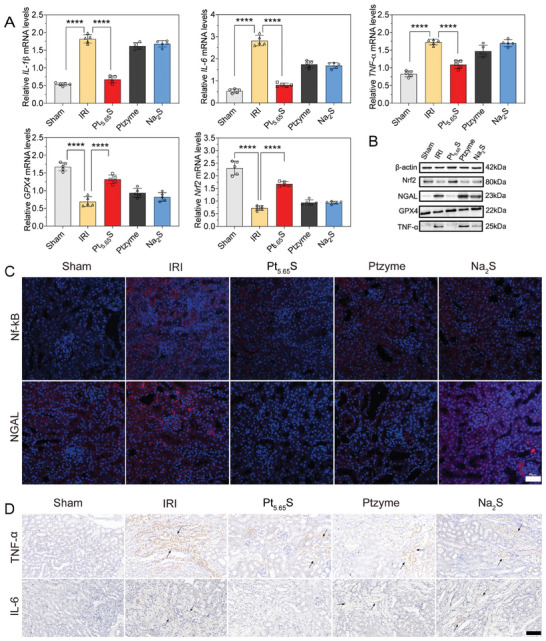
Therapeutic mechanisms of Pt_5.65_S pre‐nanozyme on IRI. A) The expression of mRNA level of *IL‐1β*, *IL‐6*, *TNF‐α*, *GPX4*, and *Nrf2* in IRI mice after different treatments. B) Western blotting of NGAL, Nrf2, GPX4, and TNF‐α protein in each group and quantitative protein expression analysis. C) Immunofluorescence staining with NF‐κB and NGAL in IRI mice after different treatments. Scale bar = 50 µm. D) Immunohistochemical staining with TNF‐α, and IL‐6 (all brown) in IRI mice after different treatments. Scale bar = 100 µm. The black arrow indicates the site of inflammation and damage in the kidneys. The outcomes were compared via one‐way ANOVA (with Tukey's post hoc correction for multiple comparisons). *n* = 5, data represent means ± SD, ns represents no statistical difference, ^***^p < 0.001, ^****^p < 0.0001.

Based on the sequencing data presented in Figure 7, we further investigated the impact of Pt_5.65_S pre‐nanozyme on the expression of genes related to the Nrf2 pathway in renal IRI mice using qPCR and western blot. Consistent with the qPCR analysis (**Figure** **8**A), the expression of Nrf2, along with its associated protein GPX4, was notably upregulated in the kidneys of Pt_5.65_S pre‐nanozyme‐treated IRI mice (Figure 8B; Figure S10, Supporting Information). It is known that the activation of the Nrf2 pathway can suppress the proinflammatory signaling pathway NF‐κB and prevent the production of inflammatory cytokines. Therefore, we determined the expression levels of these related genes. Immunofluorescence staining results indicated that Pt_5.65_S pre‐nanozyme significantly suppressed the expression of NF‐κB in IRI mice. We further measured the relative expressions of major inflammatory cytokines *IL‐6*, *IL‐1β*, and *TNF‐α* to confirm the protective effects of Pt_5.65_S pre‐nanozyme on the kidney (Figure 8C,D). These results highlight the role of Pt_5.65_S pre‐nanozyme in activating Nrf2 to protect against ROS, thereby exerting anti‐inflammatory effects for renal cell protection in renal IRI.

### 2.6 Therapeutic Efficacy of Pt_5.65_S Pre‐Nanozyme in Cisplatin‐Induced AKI

**Figure 9:**
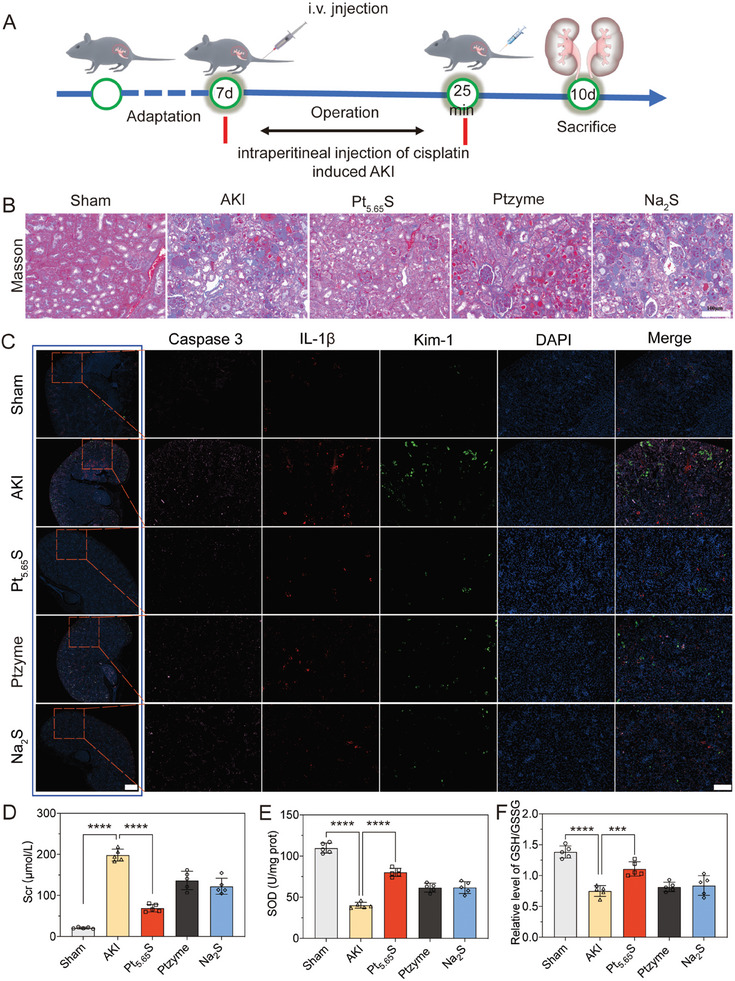
The therapeutic efficacy of Pt_5.65_S pre‐nanozyme in AKI mice models. A) Schematic diagram of the operation of cisplatin‐induced AKI in mice. B) Masson staining of kidney tissues from different group. Scale bar = 100 µm. C) Immunofluorescence staining of Caspase‐3, IL‐1β, and Kim‐1 in AKI mice after different treatments. Scale bar = 1 mm (inside frame), and Scale bar = 200 µm (out of frame). D) The level of serum creatinine (Scr) in the serum of each group. E) The levels of SOD and GSH/GSSG F) analyses in the kidneys from AKI mice after different treatments. The results were analyzed using one‐way ANOVA (with Tukey's post hoc correction for multiple comparisons), and *n* = 5, where data represents means ± SD, ns represents no statistical difference, ^***^p < 0.001, ^****^p < 0.0001.

Cisplatin is a common anticancer drug used to treat solid tumors; however, it is a significant contributor to AKI in cancer patients. Oxidative stress is a primary pathological factor that exacerbates the progression of AKI. Upon entering tubular cells, cisplatin induces oxidative stress, inflammation, cell apoptosis, and necrosis, leading to renal failure induced by cisplatin. This is primarily due to the inhibition of antioxidant enzymes such as SOD, CAT, and GSH, activation of apoptosis in renal tubules, and excessive ROS production caused by mitochondrial and DNA damage. To assess the therapeutic impact of Pt_5.65_S pre‐nanozyme on AKI in vivo, we established a mouse model of cisplatin‐induced AKI (**Figure** **9**A). Treatment with Pt_5.65_S pre‐nanozyme resulted in a significant reduction of kidney damage, as evidenced by Masson staining (Figure 9B) and immunofluorescence labeling of Kim‐1 in kidney tissues (Figure 9C). Additionally, the decreased expression of Caspase‐3 (Figure 9C) suggested the effectiveness of Pt_5.65_S pre‐nanozyme in mitigating renal tubular apoptosis. Furthermore, Scr levels were significantly lower in the Pt_5.65_S pre‐nanozyme group in comparison to the cisplatin group (Figure 9D), indicating the protective effect of Pt_5.65_S pre‐nanozyme on kidney function. Importantly, the Pt_5.65_S pre‐nanozyme group demonstrated higher levels of kidney SOD and GSH/GSSG, along with lower MDA levels compared to the AKI group (Figure 9E,F; Figure S11, Supporting Information).

**Figure 10:**
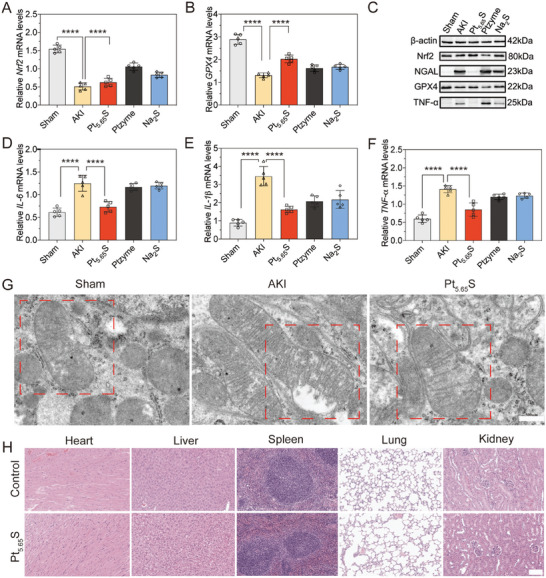
Antioxidative activity of Pt_5.65_S pre‐nanozyme in the acutely injured kidney. A) Expression of mRNA levels of *Nrf2* in AKI mice after different treatments. B) Expression of mRNA levels of *GPX4* in AKI mice after different treatments. C) Expression of NGAL, Nrf2, GPX4, and TNF‐α proteins in each group. D–F) Expression of mRNA levels of *IL‐6*, *IL‐1β* and *TNF‐α* in AKI mice after different treatments. G) TEM images of mitochondria in the kidney from each group. The red box in the figure depicts representative TEM images showing the morphology of mitochondria under different conditions. Scale bar = 500 µm. H) HE staining of the heart, liver, spleen, lung, and kidney. Scale bar = 100 µm. The outcomes were compared using one‐way ANOVA (with Tukey's post hoc correction for multiple comparisons). *n* = 5, data represent means ± SD, ns represents no statistical difference, ^****^p < 0.0001.

The results of this study demonstrate that the high therapeutic efficacy of Pt_5.65_S pre‐nanozyme against kidney injury was primarily due to the activation of the Nrf2 signaling pathway in IRI. To further explore whether Pt_5.65_S exerted a protective effect through the same mechanism in cisplatin‐induced AKI, we assessed the expression levels of Nrf2 and GPX4. Consistently, treatment with Pt_5.65_S upregulated both mRNA and protein levels of Nrf2 and GPX4 in AKI mice (**Figure** **10**A–C; Figure S12, Supporting Information). Similarly, in mouse models of cisplatin‐induced AKI, Pt_5.65_S pre‐nanozyme significantly reduced the mRNA levels of inflammatory‐related cytokines *IL‐6*, *IL‐1β*, and *TNF‐α* (Figure 10D–F). Moreover, Pt_5.65_S pre‐nanozyme treatment remarkably restored mitochondrial morphology that was damaged by AKI (Figure 10G).

To assess the biosafety of Pt_5.65_S pre‐nanozyme, injections were administered daily to male C57BL/6J mice for seven days. Hearts, livers, spleens, and lungs were extracted from each group and subjected to H&E staining. The results indicate that the Pt_5.65_S pre‐nanozyme exhibited no discernible side effects on the major organs, demonstrating its high biosafety for in vivo applications (Figure 10H).

Collectively, these in vitro and in vivo findings demonstrate that pH‐activated Pt_5.65_S pre‐nanozyme not only mimics natural antioxidant systems by functioning as CAT, SOD, and GPX4 but also releases H_2_S gas in inflammatory and acidic environments, effectively alleviating kidney injury in both the IRI and cisplatin‐induced AKI models.

## 3 Conclusion

This study presents a pioneering glomerulus‐pass and pH‐activatable pre‐nanozyme, denoted as Pt_5.65_S pre‐nanozyme. The as‐prepared pre‐nanozyme exhibits the capability to restore CAT/SOD‐mimic activities, while concurrently generating endogenous small molecules H_2_S with inflammatory acidic microenvironments. Both in vitro and in vivo assessments underscore the robust antioxidant activity of Pt_5.65_S pre‐nanozyme, effectively mitigating inflammation‐related kidney disease by attenuating the detrimental effects of ROS/RNS and reinforcing antioxidant effects through interactions with endogenous gas small molecules. The small size of the Pt_5.65_S pre‐nanozyme enables efficient elimination through the glomerulus. Moreover, the study also highlights the remarkably low biological toxicity of the pre‐nanozyme, indicating its high potential for biomedical applications. The Pt_5.65_S pre‐nanozyme offers a promising precision catalytic therapy approach for renal IRI and cisplatin‐induced AKI mice by encouraging Nrf2 activation and reducing oxidative damage, in a pH‐responsive manner. Overall, the Pt_5.65_S pre‐nanozyme represents a significant augmentation to nanozyme technology, offering a universal and safe approach to the efficient application of nanozymes in diverse biomedical contexts.

## 4 Experimental Section

The detailed experimental processes are available in the Supplementary Information.

## Conflict of Interest

The authors declare no conflict of interest.

## Supporting information

Supporting Information

## Data Availability

The data that support the findings of this study are available on request from the corresponding author. The data are not publicly available due to privacy or ethical restrictions.
