# Supplementary material for: pH‐Activatable Pre‐Nanozyme Mediated H2S Delivery for Endo‐Exogenous Regulation of Oxidative Stress in Acute Kidney Injury
Source: Adv Sci (Weinh). 2024 Mar 6;11(18):2303901. doi: 10.1002/advs.202303901 (PMC11095207; doi:10.1002/advs.202303901)
Supplement: Supplementary file 1 — Supporting Information [file ADVS-11-2303901-s001.pdf]

ADVANCED SCIENCE
Open Access

Supporting Information

for *Adv. Sci.*, DOI 10.1002/advs.202303901

pH-Activatable Pre-Nanozyme Mediated H_2S Delivery for Endo-Exogenous Regulation of Oxidative Stress in Acute Kidney Injury

Wei Jiang, Xinyue Hou, Yuanbo Qi, Zhigang Wang, Ying Liu, Xuejiao J. Gao, Tingting Wu, Jiancheng Guo, Kelong Fan and Wenjun Shang**

Supporting Information

pH-Activatable Pre-Nanozyme Mediated H_2S Delivery for Endo-Exogenous Regulation of Oxidative Stress in Acute Kidney Injury

Wei Jiang#, Xinyue Hou#, Yuanbo Qi#, Zhigang Wang, Ying Liu, Xuejiao J. Gao, Tingting Wu, Jiancheng Guo, Kelong Fan, Wenjun Shang**

Dr. W. Jiang., Miss. X. Hou., Dr. Y. Qi., Dr. Z. Wang., Pro. J. Guo., Prof. W. Shang.
Department of Kidney Transplantation, The First Affiliated Hospital of Zhengzhou University, Zhengzhou, China.
Email: fccshangwj@zzu.edu.cn

Miss. T. Wu, Dr. Y. Liu., Dr. W. Jiang, and Pro. K. Fan.
Nanozyme Medical Center, School of Basic Medical Sciences, Academy of Medical Science, Zhengzhou University, Zhengzhou 450001, China.

Dr. X. J. Gao
College of Chemistry and Chemical Engineering, Jiangxi Normal University, Nanchang 330022, P. R. China.

Prof. K. Fan.
CAS Engineering Laboratory for Nanozyme, Key Laboratory of Protein and Peptide Pharmaceuticals, Institute of Biophysics, Chinese Academy of Sciences, Beijing 100101, China.
University of Chinese Academy of Sciences, Beijing 101408, China.
Email: fankelong@ibp.ac.cn

Experimental Section

Materials.

The human embryonic kidney 293 (HEK293) cell line was purchased from iCell Bioscience Inc, Shanghai. The HEK293 cells were cultured in Dulbecco's modified Eagle's medium (DMEM) supplemented with 10% fetal bovine serum (FBS), 100 μg mL^{-1} streptomycin, and 100 U mL^{-1} penicillin at 37°C in an incubator supplied with a humidified atmosphere of 5% CO_2.

Characterizations.

The morphology of $Pt_{5.65}S$ pre-nanozyme was observed by TEM (HT7700, Hitachi, Japan). The hydrodynamic size and zeta potential of nanozymes were determined by Nanosizer Pro (Malvern Instruments, Britain). ESR spectroscopy was carried out by a ESR spectrometer (A300-10/12, Bruker, Germany), 2,2-dimethyl-1-oxido-3,4-dihydropyrrol-1-ium (DMPO) and Triacetonamine (TEMP) were used as superoxide radical and singlet oxygen catching agent, respectively. XPS was carried out by XPS (Thermo Scientific K-Alpha, USA). XRD spectroscopy was carried out by a XRD spectrometer (Bruker D8 Advance, Bruker, Germany)

Preparation of $Pt_{5.65}S$ pre-nanozyme. In a typical procedure, 133 mg PVP powders (133 mg) were dissolved in 180 mL methanol under stirring, and then a 6.0 mM certain H_2PtCl_6 aqueous solution (6.0 mM, 20.0 mL) was added dropwise, followed by continuously stirring for several minutes. After being heated to reflux for 3 h, the PVP-stabilized Ptzyme were formed. Then rotary evaporation was utilized to remove most of the solvent (methanol and water). After collecting and washing over 10 times to remove excess free PVP, the obtained samples were collection through freeze-drying. Ptzyme (10 mg) and Na_2S (1 mg) were dissolved into 10 mL of water with continuous stirring overnight, dialyze to remove free Na_2S, lyophilize and collect the obtained sample $Pt_{5.65}S$ pre-nanozyme.

SOD-like activity of nanozymes. The SOD-like activity of nanozymes was determined by analyzing the $O_2^{\bullet-}$ elimination rate of nanozymes. $O_2^{\bullet-}$ was produced by the xanthine-xanthine oxidase (X-XOD) system, and cytochrome C was used as $O_2^{\bullet-}$ indicator. The $O_2^{\bullet-}$ concentration of the control system (3 mL in total: xanthine 0.5 mL, cytochrome C 0.5 mL, xanthine oxidase 0.2 mL, PBS buffer: 1.8 mL) was detected by measuring the increase in the absorbance at 550 nm (ΔA1) of cytochrome C by ultraviolet spectrophotometer (TuxiT6, Beijing) in 1 min. When ΔA1 was adjusted to 0.025, nanozymes were added into the control system, respectively, and the increase in the absorbance at 550 nm (ΔA2) of cytochrome C in 1 minute was recorded. The $O_2^{\bullet-}$ elimination rate of nanozymes was calculated as (ΔA1-ΔA2) / ΔA1*100%.

CAT-like activity of nanozymes. The CAT-like activity of nanozymes was measured by monitoring the increase in O_2 concentration in 0.3% H_2O_2 solution using a Dissolved Oxygen Meter (InPro 6860i, Mettler Toledo, Switzerland). The reaction system contains 40 μg nanozyme and 0.3% H_2O_2 in PBS buffer. The

decomposition of H_2O_2 by nanozymes was measured by monitoring the decrease in the absorbance at 240 nm of H_2O_2.

Density functional theory (DFT) calculations of $Pt_{5.65}S$ pre-nanozyme: All the density functional theory (DFT) calculations were performed applying the Vienna Ab initio Simulation Package (VASP). We performed an all-atom optimization for the Pt(111) surface with four atomic layers. For the intermediates, we fixed the lower three Pt atomic layers to describe the bulk phase and optimized only the top layer of Pt atoms and the corresponding small molecules. The projector-augmented wave pseudopotential1-3 with Perdew, Burke and Ernzerhof (PBE) exchange-correlation functional were applied. The calculation accuracy was controled by seting the energy cutoff as 500 eV, and the convergence thresholds for the electronic structure and forces as 10 - 5 eV and 0.02 eV/Å, respectively. Standard K-space Monkhorst-Pack grid samplings4 were employed at 3 × 3 × 1. The vdW corrections in the D3BJ form 5 - 7 were also considered.

***In vitro* ROS scavenging using $Pt_{5.65}S$**

The cytotoxicity of $Pt_{5.65}S$ was determined by the CCK8 assay in vitro. Briefly, HEK293 cells were seeded into 96 well culture plates at the density of 1×10^4 cells per well and incubated at 37°C in an incubator with 5% CO_2 for 24 h. Afterward, the cell culture medium was aspirated and fresh culture media containing various concentrations of $Pt_{5.65}S$ (0 ~ 200 μg mL^{-1}) were added. After 24 h incubation, cells were gently washed once with sterile PBS, treated with 100 μL fresh culture medium and 10 μL CCK8 solution, and further incubated at 37°C for 2 h. The cell viability was then quantified by measuring the absorbance value at 450 nm with a microplate reader.

To investigate the ROS scavenging ability of $Pt_{5.65}S$ in cells, HEK293 cells were seeded into 96 well plates at the density of 1×10^4 cells per well. After 24 h incubation, $Pt_{5.65}S$ with different concentrations (0 ~ 40 μg mL^{-1}) were added to each group of wells and incubated for 60 min. Then, cells were added with 200 μM H_2O_2 and further incubated at 37°C for 24 h. Cells seeded in 96 well plates were incubated with cell counting kit-8 (CCK8, Dojindo, Japan) to detect cell viability. Wells without the addition of H_2O_2 were regarded as the negative control.

Next, cells seeded in 6-well plates were stained with Annexin V-FITC apoptosis detection kit (A211-01, Vazyme, China) to detect the ratio of apoptotic and necrotic cells. Briefly, HEK293 cells in a well were collected, washed with cold PBS, and re-suspended in 195 μL binding buffer after incubation with H_2O_2 for 24 h. Then, 10 μL Annexin V-FITC and 10 μL PI were sequentially added to the cell suspension and incubated at room temperature in the dark for 15 min. After that, cells were analyzed by the Flow cytometer (NovoCyte, Agilent, USA). At least 50,000 cells were analyzed in each sample.

Detection of Intracellular ROS

Intracellular ROS measurement was assayed by 2',7'-dichlorofluorescin diacetate (DCFH-DA) staining. HEK293 cells were incubated in confocal microscope dishes for 24 h, and the cell density was 1×10^5 cells

well^{-1}. After incubation, the cells were incubated with H_2O_2, Ptzyme, Na_2S or $Pt_{5.65}S$ pre-nanozyme + H_2O_2 for a further 24 h. The cells were then washed three times with PBS and cultured with DCFH-DA (5 μM) at 37°C for a further 30 min. Confocal laser scanning microscopy determined intracellular ROS levels (λex = 480 nm, λem = 525 nm).

Mitochondrial membrane potential detection

The mitochondrial membrane potential (MMP) of HEK293 cells was detected with JC-1 (Bryotime C2006). Briefly, HEK293 cells were seeded on sterile polylysine-coated dishes and treated with H_2O_2 for 24 h with or without $Pt_{5.65}S$. Then, the cells were incubated with JC-1in the dark for 30 min at 37°C and washed three times with PBS. Nuclei were counterstained with Hoechst 33342, and fluorescence images were captured by confocal microscopy.

Assessment of lipid peroxidation by BODIPY 581/591 C11 staining

HEK293 cells treated with or without $Pt_{5.65}S$ for 24 h were incubated with 2 μM BODIPY 581/591 C11 (D3861, Thermo Fisher Scientific) for 40 min at 37°C. The stained cells were analyzed using an confocal microscope (Carl Zeiss). Stock solutions of BODIPY 581/591 C11 were prepared by dissolving 2 mg BODIPY 581/591 C11 in 100 μL dimethyl formamide.

Mitochondrial fragmentation analysis

The mitochondrial fragmentation of HEK293 cells induced by H_2O_2 was determined by fluorescence imaging. Briefly, HEK293 cells (6 × 10^4 cells mL^{-1}, 2 mL) were seeded in 35 mm confocal dishes with coverglass bottom and attached for 24 hours. The H_2O_2-damaged cells were further incubated with or without $Pt_{5.65}S$ for another 24 hours. Then the cells were stained with Hoechst 33342 (blue) and MitoTracker (red), then incubated in an incubator for 30 minutes. After rinsing three times with cold PBS, the cells were examined on mitochondrial morphology under a confocal microscope.

Measurement of malondialdehyde (MDA), GSH, and GSSG

The levels of MDA in the kidney tissues were detected by using a lipid peroxidation MDA assay kit (Beyotime; S0131) according to the manufacturer's instructions. A commercially available GSH and GSSG assay kit (Beyotime, China, S0053) was used to measure the levels of reduced glutathione (GSH) and oxidized glutathione disulfide (GSSG) in the kidney tissues and HEK293 cells. Briefly, tissues or cultured cells were collected, washed with ice-cold PBS three times, and lysed in lysis buffer; the supernatant was collected and used for GSH and GSSG detection.

Measurement of SOD-like activity and CAT-like activity

The SOD-like activity of kidney tissues and HEK293 cells was determined by formazan formation using a SOD assay kit (WST-8 method, Beyotime S0101S). According to the manufacturer's instructions, WST-8 can react with the superoxide anion ($O_2^{\bullet-}$) catalyzed by Xanthine Oxidase (XO) to produce water-soluble dirty dye (formazan dye). Because SOD can catalyze the disambiguation of superoxide anion, this reaction

step can be suppressed by SOD. Therefore, the activity of SOD is negatively correlated with the amount of dirty dye production, so the enzyme activity of SOD can be calculated by colorimetric analysis of WST-8 products.

The CAT-like activity of kidney tissues and HEK293 cells was determined by formazan formation using a CAT assay kit (Beyotime S0051). According to the manufacturer's instructions, when hydrogen peroxide is relatively abundant, catalase can catalyze hydrogen peroxide to produce water and oxygen. Under the catalysis of Peroxidase, the residual hydrogen peroxide can oxidize the color substrate and produce the red product (N-(4-antipyrine)-3-chloro-5-sulfonate-p-benzoquinonemonoimine). The maximum absorption wavelength is 520nm. With the hydrogen peroxide standard, a standard curve can be made to calculate how much catalase in the sample catalyzed the conversion of hydrogen peroxide into water and oxygen per unit time per unit volume, and thus the catalase activity in the sample can be calculated.

Quantitative real-time PCR (qRT-PCR)

TRIzol (Solarbio) was used to extract total RNA from kidney tissues or cultured cells according to the manufacturer's instructions. One microgram of RNA sample was reverse transcribed using a reverse transcriptase kit (Vazyme, Nanjing, R232-01). The primers were designed and synthesized by Sangon Biotech (Shanghai, China). Messenger RNA (mRNA) expression was measured by qRT-PCR by a QuantStudio 3 real-time PCR system (Applied Biosystems, Foster City, CA, USA) using SYBR Green master mix (Vazyme, Nanjing, China; q111-02/03). Cycling conditions of qRT-PCR were 95°C for 10 min followed by 40 cycles at 95°C for 15 s and 60°C for 1 min. The relative threshold cycle values (ΔCt) were used to analyze and calculate the relative levels of mRNA expression normalized to GAPDH that was used as an internal control; then, fold changes were estimated using the 2−ΔΔCt method to determine the relative mRNA levels.

Mouse models of IRI

All animal experiments were approved by the Institution Animal Ethics Committee of Zhengzhou University (license No. ZZU-LAC20200703[06]). An IRI rat model was established by the ischemia/reperfusion method. Male C57BL/6J mice (20 ~ 25 g) were anesthetized, shaved, and disinfected. The renal pedicle was exposed by a lumbodorsal incision, after which the renal arteries were carefully separated from both kidneys. The renal artery was quickly clamped with an arterial clamp for 30 min at room temperature. After 30 min of ischemia, the arterial clamp was released to restore blood flow. After the successful establishment of the IRI models, the mice were randomly assigned to the following groups: i. IRI, ii. IRI + Ptzyme (15 mg kg^{-1}, *i.v.*); iii. IRI + Na_2S (15 mg kg^{-1}, *i.v.*); iv. IRI + $Pt_{5.65}S$ (15 mg kg^{-1}, *i.v.*) (five mice in each group). Sham-operated mice were used as controls.

Then, animals were sacrificed. Serum samples were obtained to determine the levels of serum creatinine (Scr), measurement of malondialdehyde (MDA), GSH/GSSG, and blood urea nitrogen (BUN) for the

evaluation of renal and liver function. Additionally, the kidneys and other organs were embedded in paraffin and sectioned for subsequent tissue staining experiments.

Mouse models of AKI

Male C57BL/6J mice aged 6 ~ 8 weeks were selected, and all mice were received intraperitoneal injections of cisplatin (30 mg kg^{-1}). 3 days after injections, the mice in each group were sacrificed to monitor the model development. The blood samples and renal tissues were obtained and analyzed. The mice were intravenously injected with Ptzyme, Na_2S, and $Pt_{5.65}S$ (15 mg kg^{-1} in 100 μL PBS) at 1 h after intraperitoneal injections of cisplatin for the treatment group.

Western blot

Renal tissues were homogenized and lysed in modified RIPA lysis buffer at 4°C. Protein concentrations were determined by the BCA protein assay. Approximately, 25 μg protein samples were separated in 12% sodium dodecyl sulfate-polyacrylamide gel electrophoresis and electrophoretically transferred onto PVDF membranes. The membranes were blocked, and incubated with primary antibodies, including NGAL (Abcam, ab216462, 1:1000), NRF2 (Abcam, ab92946, 1:1000) TNF-α (Abcam, ab123818, 1:1000), GPX4(Proteintech, 67763-1-Ig, 1:1000) and β-actin (Abcam, ab6276, 1:5000) overnight at 4°C. After that, the blots were washed and incubated with the secondary HRP-conjugated anti-rabbit IgG antibody (1:5000) or anti-mouse (1:5000) for 1h at room temperature. The membrane was then exposed to Amersham prime ECL Plus detection system (Pittsburgh, PA) after which chemiluminescence detection was performed.

RNA sequencing

Mice were sacrificed to harvest the kidneys. The kidneys were washed thrice with saline and snap-frozen in liquid nitrogen. Total RNA was extracted by using the TIANGEN Animal Tissue Total RNA Extraction Kit (DP419, TIANGEN, China). RNA integrity was verified by Agilent 4200 Bioanalyzer and quantified using ND-2000 (Nanodrop Technology). Cell rRNA was removed by using an MGIEasy rRNA removal kit. Library construction was carried out using the MGIEasy RNA Library Prep Set (96 RXN) according to the manufacturer's protocol. Libraries were visualized on the Agilent 4200 Bioanalyzer to check insert size and quantified by using the Qubit Fluorometers to determine the concentration. Libraries were pooled and loaded on the flow cell to run on MGISEQ-2000 sequencer as paired-end read for 150 cycles on each side.

Statistical analysis

All statistical analyses were conducted using the GraphPad Prism 8.0.2 in a blinded manner. The differences in means between different groups were determined by one-way ANOVA Tukey's multiple comparisons test. The specific statistical method and statistical analysis results for each experiment were listed in the corresponding figure legends.

Supplementary Figures

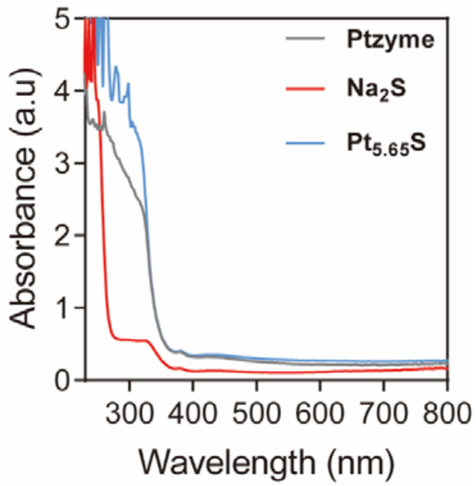

Figure S1. Ultraviolet absorption spectrum of $Pt_{5.65}S$ pre-nanozyme, Ptzyme, and Na_2S.

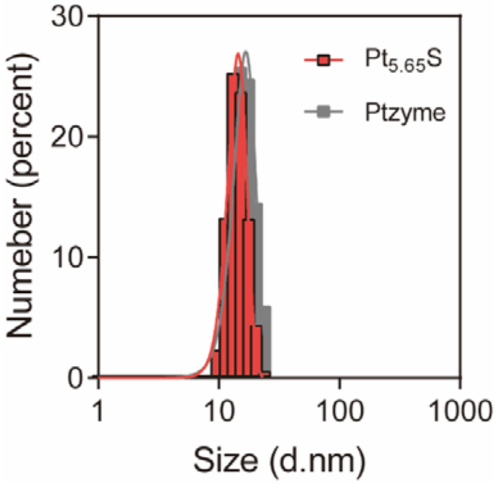

Figure S2. Hydrodynamic diameters of $Pt_{5.65}S$ pre-nanozyme and Ptzyme.

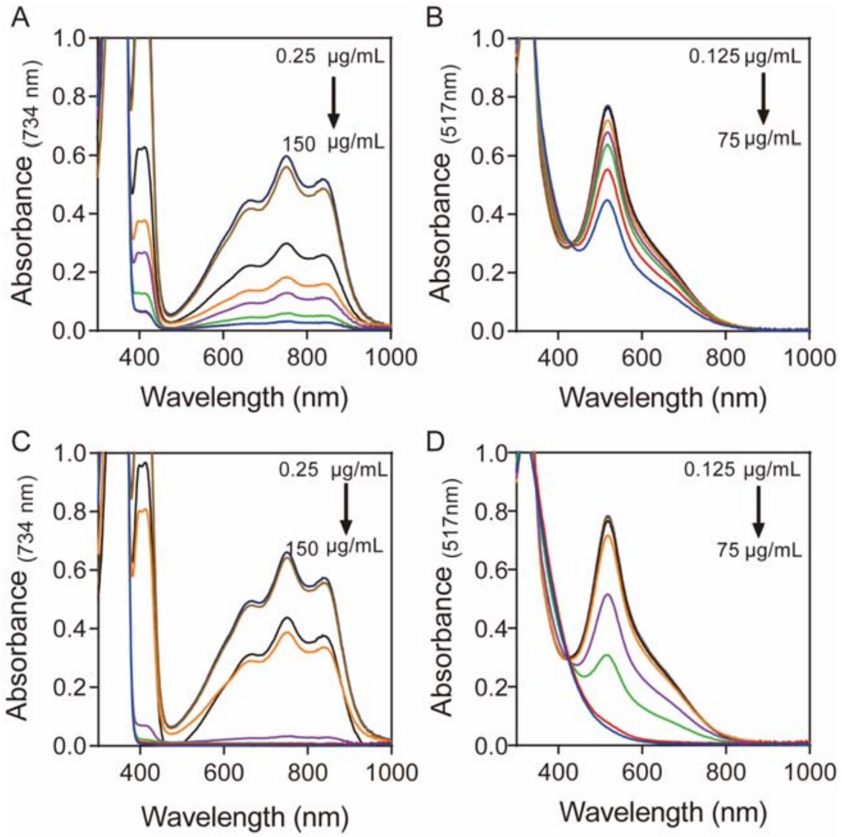

Figure S3. RNS scavenging capability analyses of Ptzyme and $Pt_{5.65}S$ pre-nanozyme. $ABTS^{\bullet +}$ spectra at 734 nm after the addition of different concentrations of Ptzyme (A) and $Pt_{5.65}S$ pre-nanozyme (C). $DPPH^{\bullet}$ spectra at 517 nm after the addition of different concentrations of Ptzyme (B) and $Pt_{5.65}S$ pre-nanozyme (D).

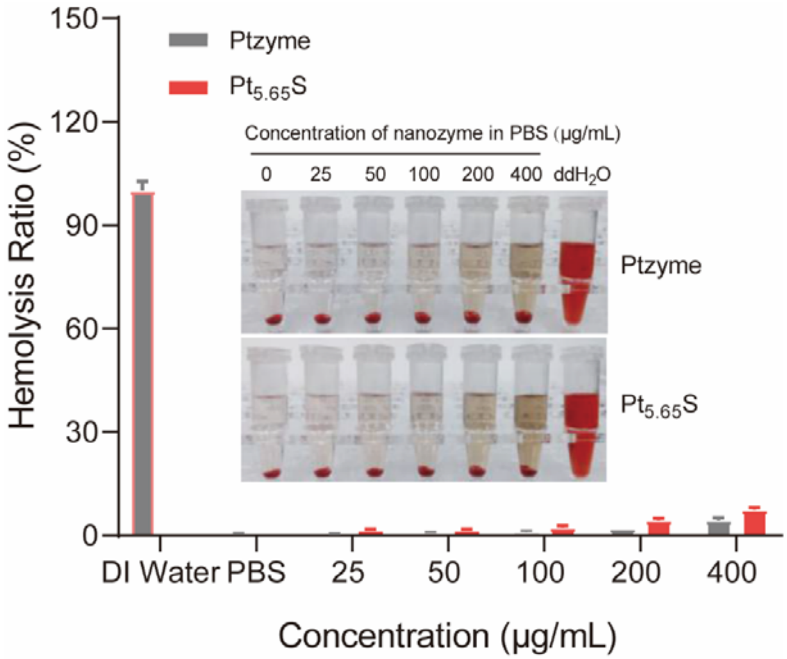

Figure S4. Hemolysis test. Comparison of hemolysis induced by PBS, ddH_2O, and different concentration of Ptzyme and $Pt_{5.65}S$ pre-nanozyme. Insert figurs are representative images demonstrating the degrees of hemolysis of Ptzyme and $Pt_{5.65}S$ pre-nanozyme.

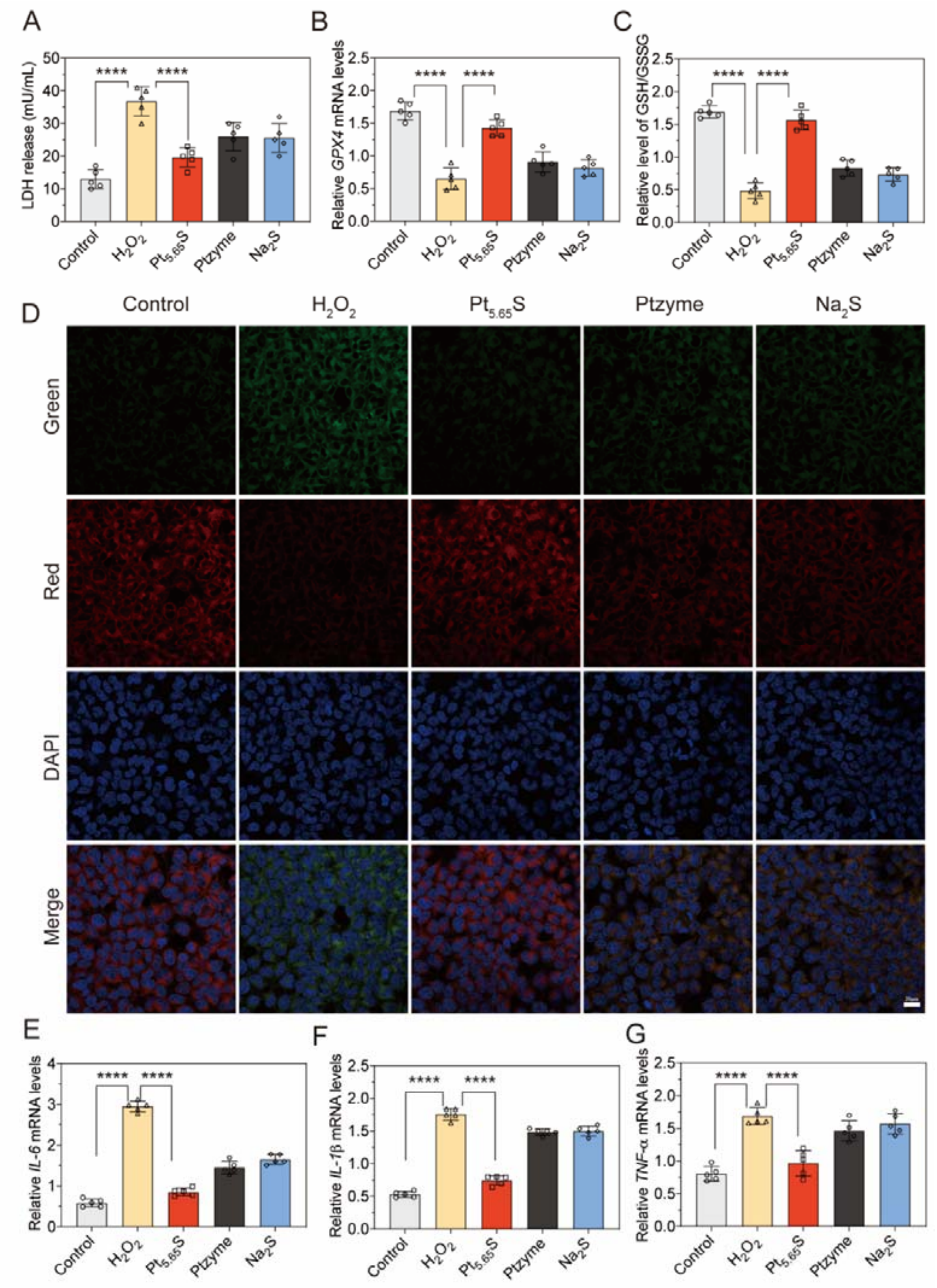

Figure S5. *In vitro* anti-apoptotic activity of $Pt_{5.65}S$ pre-nanozyme. (A) The level of LDH in H_2O_2-stimulated HEK293 cells after treatment with Ptzyme, Na_2S, and $Pt_{5.65}S$ pre-nanozyme for 24 h. (B) Relative mRNA levels of each group of *GPX4*. (C) The level of GSH/GSSG in H_2O_2-stimulated HEK293 cells after treatment with Ptzyme, Na_2S, and $Pt_{5.65}S$ pre-nanozyme for 24 h. (D) Representative micrographs show BODIPY 581/591 C11 staining in HEK293 cells (red, lipids; green, oxidized lipids). Scale bar = 20 μm. (E-G) Relative mRNA levels of each group of *IL-6*, *IL-1β*, *TNF-α*. The significance of the data was evaluated according to one-way ANOVA Tukey's multiple comparisons test. n = 5, data represent means ± SD, $****p < 0.0001$.

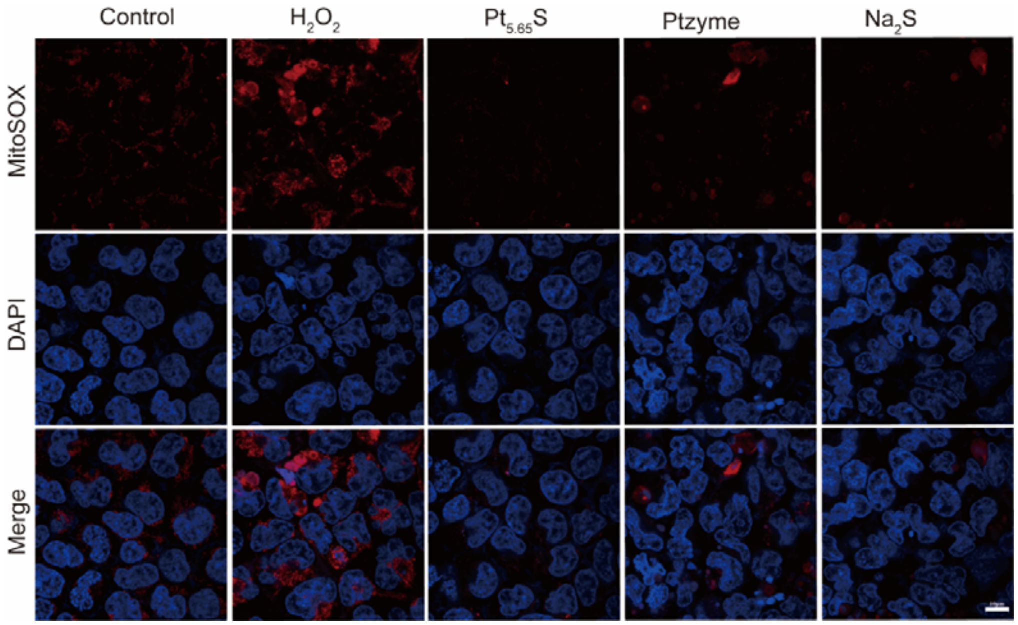

Figure S6. Representative micrographs showing MitoSOX staining in HEK293 cells. Scale bar = 10 µm.

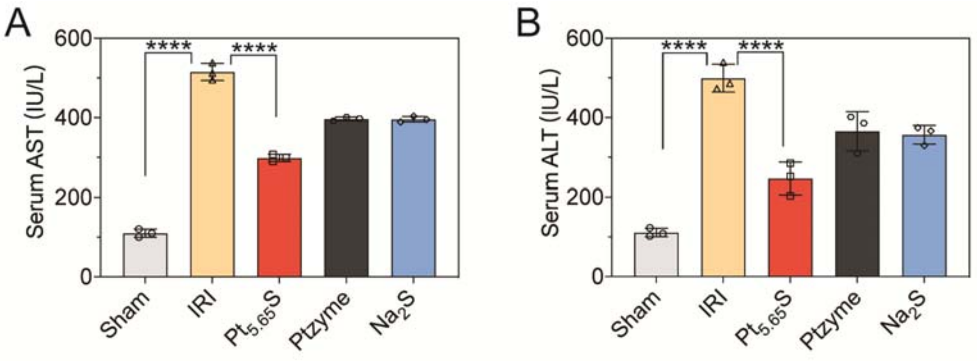

Figure S7. Quantification of liver injury with different group treatments. (A) The level of serum AST was detected in each group. (B) The level of ALT was detected in the serum of each group. The significance of the data was evaluated according to one-way ANOVA Tukey's multiple comparisons test. n = 3, data represent means ± SD, ****$p < 0.0001$.

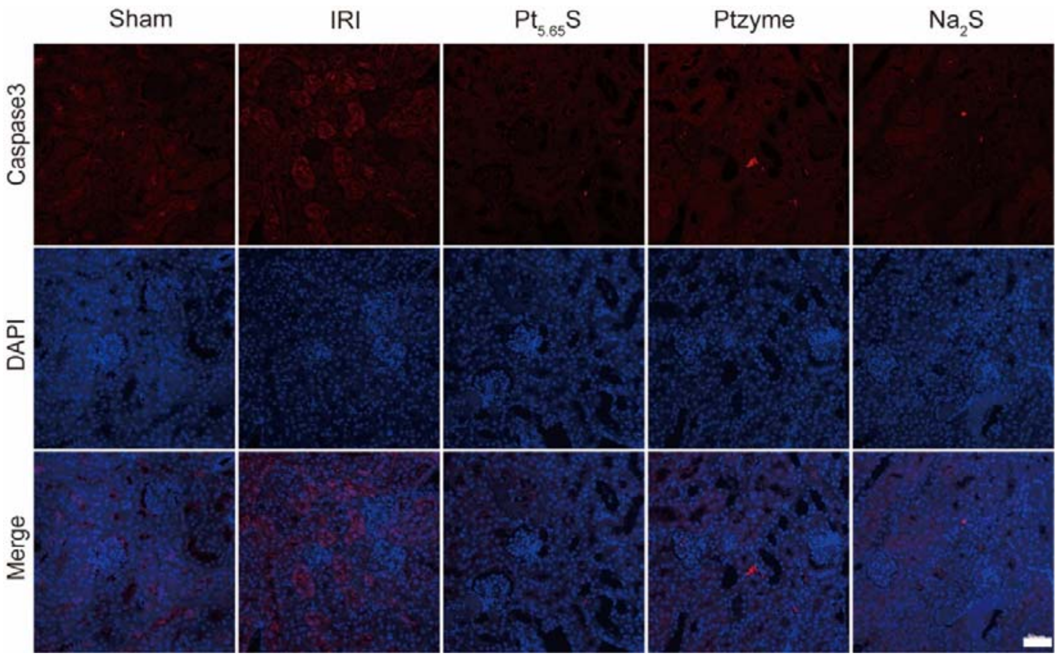

Figure S8. Immunofluorescence staining with Caspase 3 in IRI mice after different treatments. Scale bar = 50 μm.

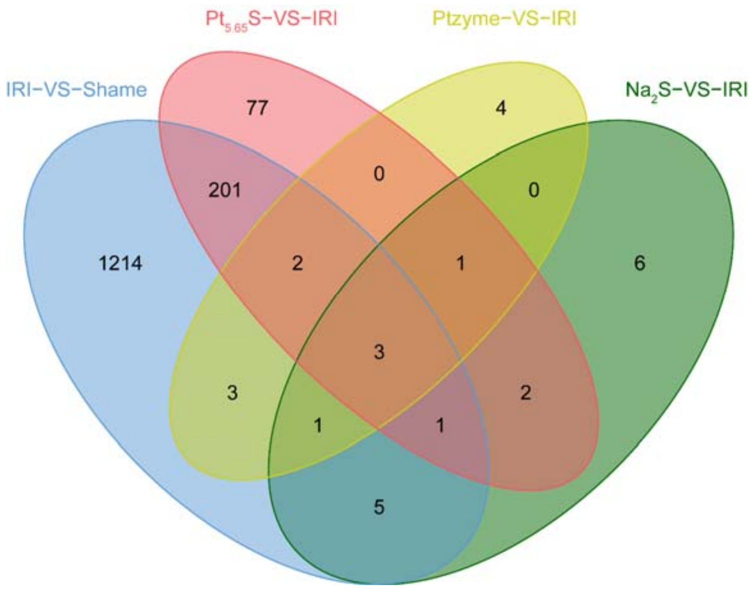

Figure S9. Venn diagram of transcriptomic profiles among the DEGs, and immune-related genes.

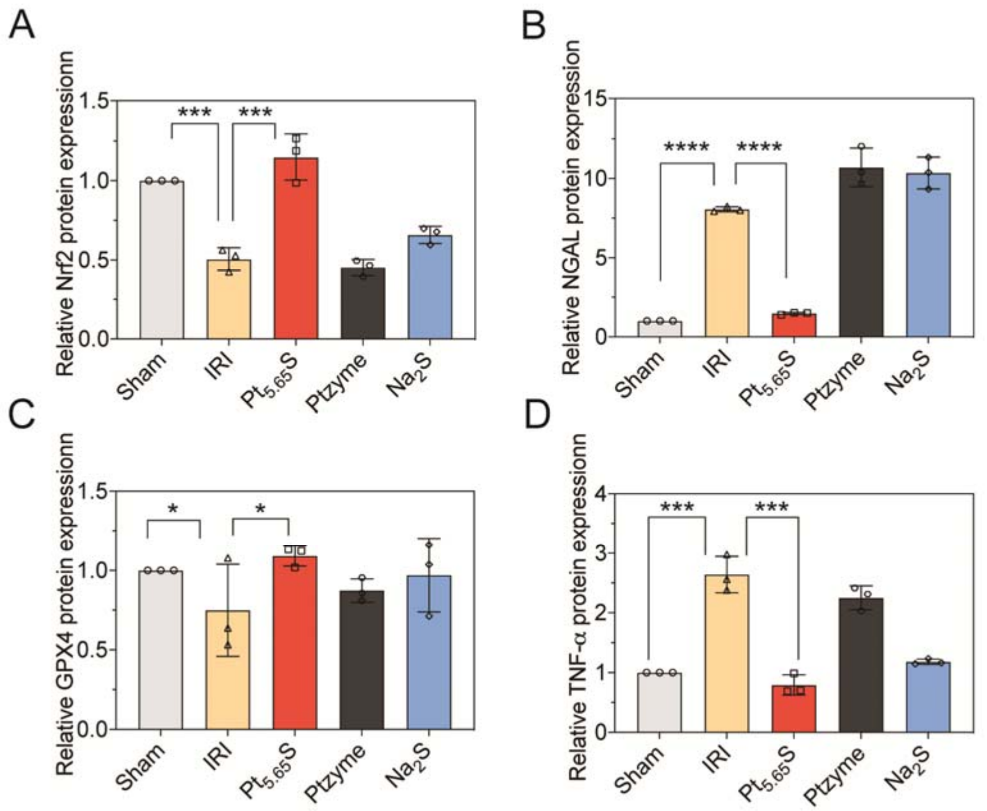

Figure S10. Quantitative analysis of relative protein expression levels in the kidney tissues. (A) Quantitative protein expression analysis showing the expression of Nrf2 in Figure 8B, β-actin served as a loading control. (B) Quantitative analysis showing the expression of NGAL, GPX4 (C), and TNF-α protein levels (D) in each group, β-actin served as a loading control. The significance of the data was evaluated according to one-way ANOVA Tukey's multiple comparisons test. n = 3, data represent means ± SD, $*p < 0.1$, $***p < 0.001$, $****p < 0.0001$.

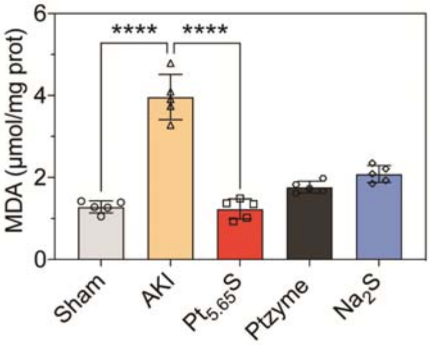

Figure S11. The level of MDA was detected in the kidney of each group. The significance of the data was evaluated according to one-way ANOVA Tukey's multiple comparisons test. n = 5, data represent means ± SD, ****$p < 0.0001$.

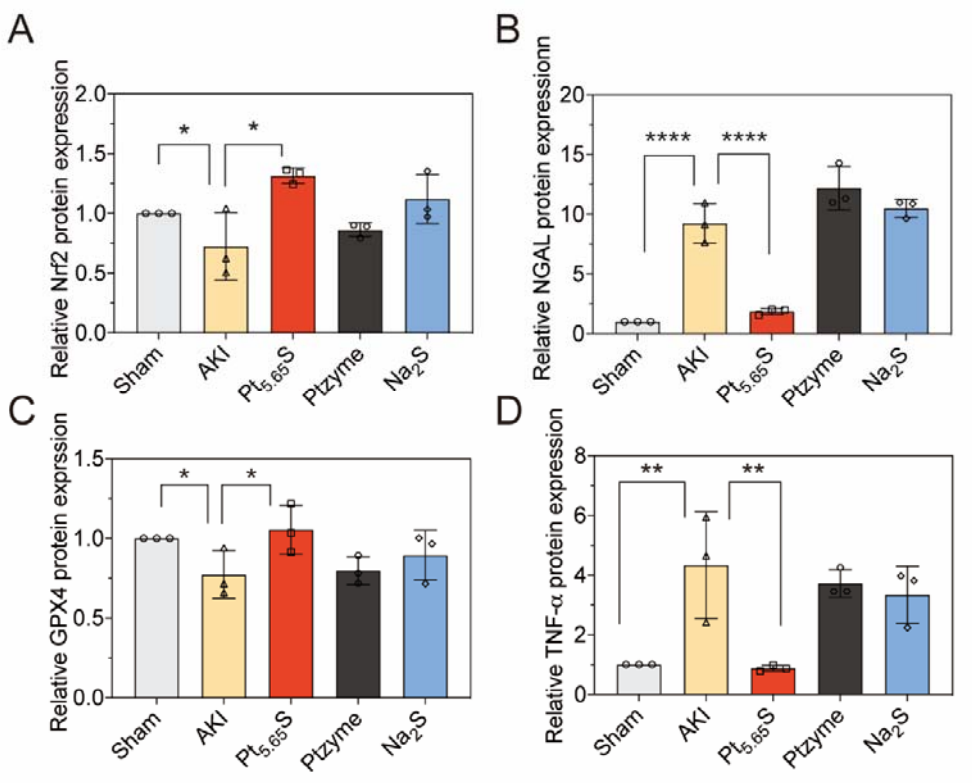

Figure S12. Quantitative analysis of relative protein expression levels in the kidney tissues. (A) Quantitative protein expression analysis showing the expression of Nrf2, NGAL (B), GPX4 (C), and TNF-α protein levels (D) in Figure 10C, β-actin served as a loading control. The significance of the data was evaluated according to one-way ANOVA Tukey's multiple comparisons test. n = 3, data represent means ± SD, $*p < 0.1$, $**p < 0.05$, $****p < 0.0001$.

Reference

1. Kresse, G.; Furthmüller, J., Efficiency of ab-initio total energy calculations for metals and semiconductors using a plane-wave basis set. *Computational materials science* **1996,** *6* (1), 15-50.

2. Kresse, G.; Joubert, D., From ultrasoft pseudopotentials to the projector augmented-wave method. *Physical Review B* **1999,** *59* (3), 1758.

3. Blöchl, P. E., Projector augmented-wave method. *Physical review B* **1994,** *50* (24), 17953.
